# PRMT1 reverts the immune escape of necroptotic colon cancer through RIP3 methylation

**DOI:** 10.1038/s41419-023-05752-w

**Published:** 2023-04-01

**Authors:** Lian Zhang, Yujiao He, Yi Jiang, Qi Wu, Yanchen Liu, Qingqiang Xie, Yuxiu Zou, Jiaqian Wu, Chundong Zhang, Zhongjun Zhou, Xiu-Wu Bian, Guoxiang Jin

**Affiliations:** 1 Medical Research Institute, Guangdong Cardiovascular Institute, Guangdong Geriatrics Institute, Guangdong Provincial People’s Hospital (Guangdong Academy of Medical Sciences), Southern Medical University, Guangzhou, 510080 China; 2 Institute of Pathology and Southwest Cancer Center, Southwest Hospital, Third Military Medical University (Army Medical University) and Key Laboratory of Tumor Immunopathology, Ministry of Education of China, Chongqing, 400038 China; 3 School of Biomedical Sciences, LKS Faculty of medicine, The University of Hong Kong, Hong Kong, China; 4 College of Basic Medical Sciences, Chongqing Medical University, Chongqing, 400016 China; 5 Department of Biochemistry and Molecular Biology, College of Basic Medical Sciences, Chongqing Medical University, Chongqing, 400016 China

**Keywords:** Cell biology, Cancer

## Abstract

Necroptosis plays a double-edged sword role in necroptotic cancer cell death and tumor immune escape. How cancer orchestrates necroptosis with immune escape and tumor progression remains largely unclear. We found that RIP3, the central activator of necroptosis, was methylated by PRMT1 methyltransferase at the amino acid of RIP3 R486 in human and the conserved amino acid R479 in mouse. The methylation of RIP3 by PRMT1 inhibited the interaction of RIP3 with RIP1 to suppress RIP1-RIP3 necrosome complex, thereby blocking RIP3 phosphorylation and necroptosis activation. Moreover, the methylation-deficiency RIP3 mutant promoted necroptosis, immune escape and colon cancer progression due to increasing tumor infiltrated myeloid-derived immune suppressor cells (MDSC), while PRMT1 reverted the immune escape of RIP3 necroptotic colon cancer. Importantly, we generated a RIP3 R486 di-methylation specific antibody (RIP3^ADMA^). Clinical patient samples analysis revealed that the protein levels of PRMT1 and RIP3^ADMA^ were positively correlated in cancer tissues and both of them predicted the longer patient survival. Our study provides insights into the molecular mechanism of PRMT1-mediated RIP3 methylation in the regulation of necroptosis and colon cancer immunity, as well as reveals PRMT1 and RIP3^ADMA^ as the valuable prognosis markers of colon cancer.

## Introduction

Necrotic cell death is featured by the morphology changes of cell swelling, plasma membrane rupture and organelle breakdown, distinguished from apoptosis and other types of cell death. Necrotic cell death has been shown to facilitate cancer chemotherapy [1]. On the other hand, necrotic cells release the intracellular contents that generate damage associated molecular patterns (DAMPs), resulting in the alteration of cellular communication, cell fate decision and immune responses in the local microenvironment [2, 3]. Necrosis-driven immune remodeling is believed to promote immune evasion and tumorigenesis, largely through the myeloid-derived suppressor cells (MDSC) recruitment and T cell inhibition [4].

Necrosis was once thought of as a type of unregulated cell death resulting from physicochemical stresses, until the programmed necrosis (termed necroptosis) was unraveled [3, 5]. RIP3 (receptor interacting protein kinase 3), a RHIM (RIP homotypic interaction motif) domain-containing serine/threonine kinase, is the central regulator of cellular necroptosis pathway [6]. With the necrotic stimulation of TNF (tumor necrosis factor) along with SMAC mimetics and caspase inhibitors, RIP3 interacts with another RHIM domain kinase RIP1 (receptor interacting protein kinase 1) to form a necroptotic complex termed necrosome, resulting in RIP3 phosphorylation and activation. Activated RIP3 consequently phosphorylates MLKL (mixed lineage kinase domain-like protein) and causes necroptotic cell death [3, 7–11]. The activation of RIP3 is tightly regulated by posttranslational protein modifications. RIP3 phosphorylation is essential for the necrosome stabilization, while the cleavage of RIP3 by caspase 8 has been reported to inactivate necroptosis [3, 10, 12]. In addition, RIP3 undergoes ubiquitination at multiple sites, which play different roles of either driving RIP3 protein degradation or stabilizing necrosome [13–18]. The emerging knowledge of RIP3 protein modifications provide comprehensive insights into the regulation of necroptosis signaling.

Methylation is a ubiquitously occurred modification type that has been identified for all of DNA, RNA and proteins. DNA methylation is mainly involved in gene transcription [19], while RNA methylation is important for RNA stability and translation [20]. The protein methylation is complicated by methylating various amino acids, among which the methylation of lysine (K) and arginine (R) are mostly studied. K/R methylation regulates not only protein expression but also protein interaction and signal transduction, participating in diverse cellular functions and physiological/pathological conditions [19, 21]. A previous literature has mentioned that DNA hypermethylation prevents RIP3 transcription, which might be responsible for the low expression or absence of RIP3 in certain investigated cancer cells. In contrast, the same study has also revealed that many other cancer cells including HT29 colon cancer cells maintain high expression of RIP3 expression [1]. It is worthy of investigating how the high level of RIP3 and necroptosis in colon cancer is concisely regulated, and whether protein methylation is involved in the process.

Here we found that RIP3 protein was methylated on the amino acid of arginine 486 (R486) in human and the conserved amino acid R479 in mouse. The mutation of R486 or R479 to K (lysine) lost methylation but promoted necroptosis in both human and mouse colon cancer cells. In addition, we identified PRMT1 as the methyltransferase of RIP3. PRMT1 methylated RIP3 to inhibit necroptosis, while PRMT1 knockdown repressed RIP3 methylation to activate necroptosis. Mechanistically, the methylation of RIP3 inhibited the interaction of RIP3 with RIP1 to prevent RIP3-RIP1 necrosome complex formation, thereby inhibiting the phosphorylation of RIP3, RIP1, MLKL and consequent necrotic cell death. On the other hand, we found that R479K mutant activated RIP3 to promote immune evasion of colon cancer. In contrast, PRMT1 inhibited RIP3 to suppress cancer immune escape and tumor growth. We have also generated a specific antibody for human RIP3 R486 methylation and verified that both RIP3 protein methylation and PRMT1 protein in clinical human colon cancer samples was associated with the longer patient survival. Our study not only demonstrates a PRMT1-RIP3 axis in regulating necroptosis and tumor immune microenvironment but also reveals PRMT1 and RIP3 methylation as the molecular prognosis biomarkers of colon cancer.

## Result

### RIP3 undergoes arginine methylation

**Fig. 1:**
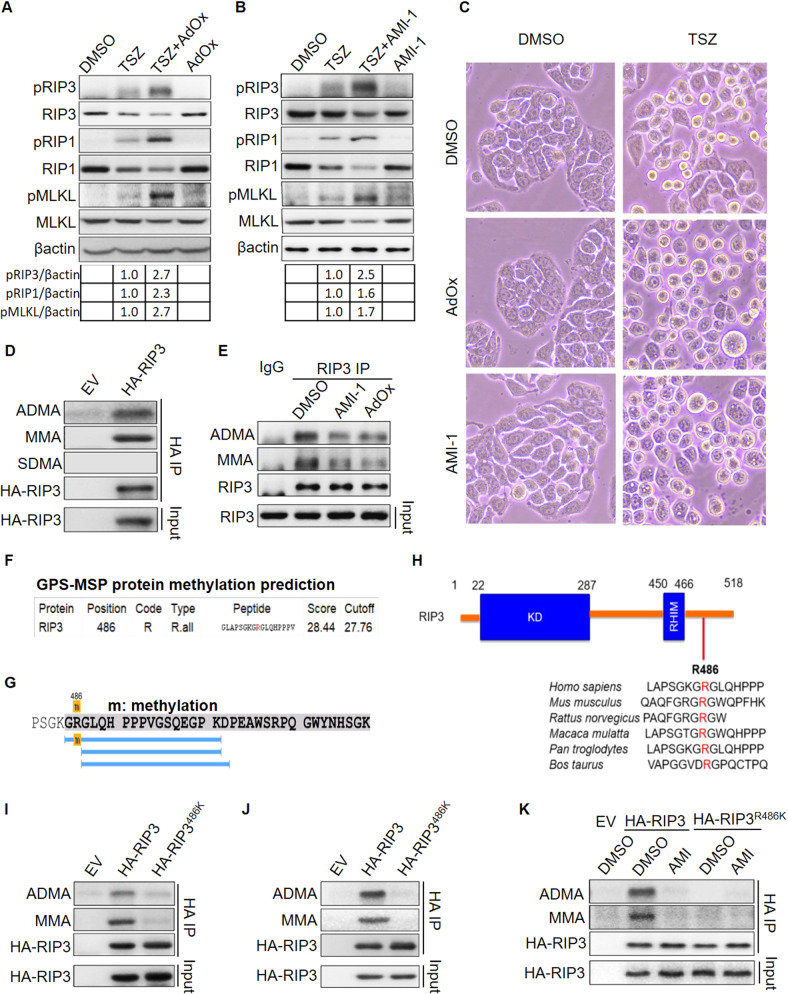
RIP3 undergoes arginine methylation at R486. **A** HT29 cells were treated with or without TSZ (20 ng/ml TNF-alpha, 100 nM LCL-161, 50 µM Z-VAD-fmk) and 25 µM AdOx (Adenosine Dialdehyde) for 6 h. The indicated necroptosis proteins were analyzed by immunoblot. **B** HT29 cells were treated with or without TSZ (20 ng/ml TNF-alpha, 100 nM LCL-161, 50 µM Z-VAD-fmk) and 25 µM AMI-1 for 6 h. The indicated necroptosis proteins were analyzed by immunoblot. **C** The morphology of HT29 cells treated with or without TSZ and AdOx (25 µM), AMI-1 (25 µM) for 8 h. **D** The ADMA, MMA, SDMA methylation of overexpressed RIP3 in HEK293T cells were analyzed by immunoprecipitation and immunoblot. **E** The ADMA and MMA methylation of endogenous RIP3 in HT29 cells treated with or without AMI-1 and AdOx inhibitors were analyzed by immunoprecipitation and immunoblot. **F** RIP3 methylation at arginine 486 was predicted with GPS-MSP protein methylation prediction software. R indicates arginine. R.all indicates all types of arginine methylation. **G** The methylation of RIP3 R486 was identified by mass spectrum. **H** Sequence alignment of the conserved amino acids of human RIP3 R486 in multiple species. **I** The methylation of transient overexpressed RIP3 and RIP3^R486^ mutant in HEK293T cells was analyzed by immunoprecipitation and immunoblot. **J** The methylation of stable overexpressed RIP3 and RIP3^R486^ mutant in HT29 cells was analyzed by immunoprecipitation and immunoblot. **K** The methylation of stable overexpressed RIP3 and RIP3^R486^ mutant in HT29 cells treated with or without AMI-1 was analyzed by immunoprecipitation and immunoblot.

Some cancer cell lines display increased DNA methylation in the genome of RIP3 to inhibit RIP3 expression and necroptotic cell death, while the studies also demonstrate that RIP3 is highly expressed in many other cancer cells including HT29 human colon cancer cell, a very commonly used cell model in studying necroptosis. The high expression of RIP3 may be independent of DNA methylation [1, 22]. We used AdOx (Adenosine Dialdehyde), an inhibitor of pan methyltransferases to treat HT29. AdOx alone indeed displayed no influence on the levels of ether total or phosphorylated RIP3, RIP1 and MLKL, supporting that the static expression of RIP3, RIP1 and MLKL was not affected by methylation (Fig. 1A). In contrast, when necroptosis of HT29 cells was activated by TSZ (TNF-alpha, LCL-161, Z-VAD-fmk), AdOx enhanced the necroptosis signal transduction as shown by the increased amount of pRIP3, pRIP1 and pMLKL (Fig. 1A). The cell morphology analysis consistently showed that AdOx increased the necroptosis in TSZ induced HT29 cells (Fig. 1C). The result suggests that methylation might suppress necroptosis independent of the expression of RIP3, RIP1 and MLKL.

As a pan methyltransferase inhibitor, AdOx inhibits both DNA methylation and protein methylation. The increased phosphorylation of RIP3, RIP3, MLKL in AdOx treated necroptotic cells led us to explore whether protein methylation contributes to necroptosis suppression. Lysine and arginine methylation are two most studied types of posttranslational protein methylation. To determine which type may be responsible for the necroptosis inactivation, we treated the cells with AMI-1, an inhibitor specific for the protein arginine methyltransferase. The same as that of AdOx, AMI-1 increased the levels of pRIP3, pRIP1, pMLKL (Fig. 1B) and the necroptotic morphology (Fig. 1C) in the TSZ-induced necroptotic cells, suggesting that the arginine methylation negatively regulated necroptosis.

Three types of methylated arginine have been described in mammalian cells, including the mono-methylarginine (MMA), asymmetric di-methylarginine (ADMA) and symmetric di-methylarginine (SDMA). ADMA and SDMA are catalyzed by type I or type II arginine methyltransferases respectively, while MMA is catalyzed by both type I and type II enzymes and is also an intermediate state of ADMA or SDMA [23]. We used the specific antibodies recognizing ADMA, SDMA and MMA respectively and identified ADMA and MMA but not SDMA methylation in overexpressed human RIP3 in HEK293T cells (Fig. 1D). In consistent with the exogenous RIP3, the endogenous RIP3 undergoes ADMA and MMA modification in HT29 cells (Fig. 1E). We further demonstrated that the methylation of RIP3 were suppressed by both AdOx and AMI-1 methyltransferase inhibitors (Figs. 1E and S1A, B).

We would like to identify the methylation site of RIP3. To this end, we performed the protein methylation prediction with the GPS-MSP methylation prediction software [24]. The result revealed arginine 486 of human RIP3 as a potential methylation site (Fig. 1F). Then mass spectrum analysis further identified the methylation site on arginine 486 (R486) (Fig. 1G), which is a conserved amino acid in the mammalian species including human and mouse (Fig. 1H). To confirm the methylation of R486 in vivo, mutant RIP3^R486K^ was constructed by mutating R486 to K(lysine) amino acid. RIP3^R486K^ was absent of ADMA di-methylation and MMA mono-methylation compared to the wild-type RIP3 in either transient overexpression HEK293T cells or stable overexpression HT29 cells (Fig. 1I, J). Arginine methyltransferase inhibitor AMI-1 abolished the methylation of RIP3 but not RIP3^R486K^ mutant (Fig. 1K).

Collectively, we demonstrate that human RIP3 undergoes the mono-methylation and asymmetric di-methylation at R486. Methyltransferase inhibitors AdOx and AMI-1 repress RIP3 methylation and promote TSZ-induced necroptosis.

### PRMT1 methylates RIP3 at R486

**Fig. 2:**
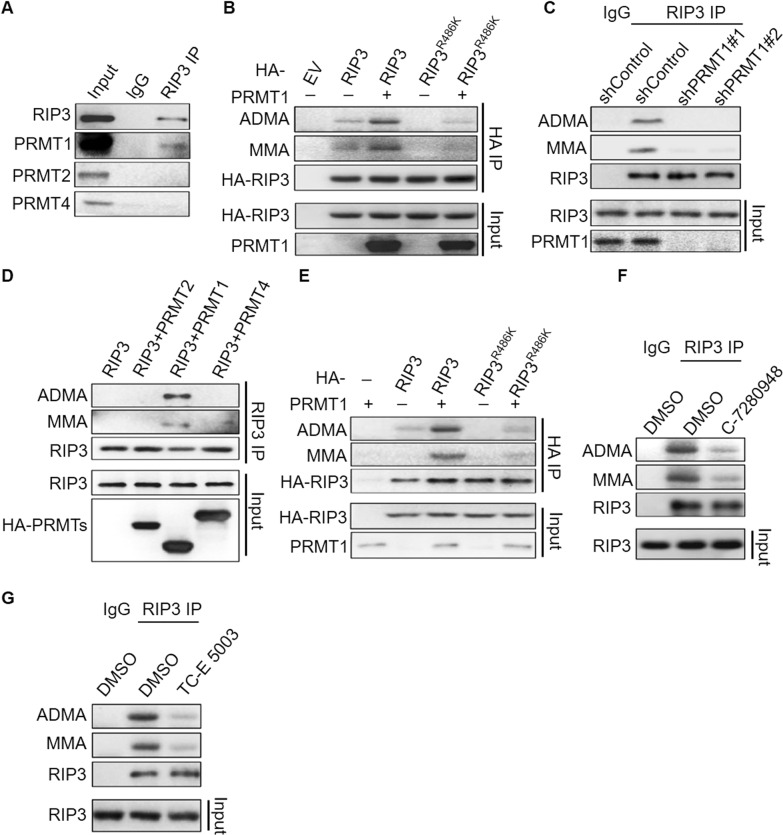
PRMT1 methylates RIP3 at R486. **A** Co-IP analysis of protein interaction between endogenous RIP3 and PRMT methyltransferases (PRMT1, PRMT2, PRMT4) in HT29 cells. **B** The methylation of stable overexpressed RIP3 and RIP3^R486^ in HT29 cells with or without PRMT1 overexpression was analyzed by immunoprecipitation and immunoblot. **C** The methylation of endogenous RIP3 in the control and PRMT1 stable knockdown HT29 cells was analyzed by immunoprecipitation and immunoblot. **D** In vitro methylation of commercial recombinant RIP3 by PRMT1. **E** In vitro methylation of purified RIP3 and RIP3^R486^ by PRMT1. **F** The methylation of endogenous RIP3 in the HT29 cells treated with or without PRMT1 inhibitor C-7280948 (100 nM) for 6 h was analyzed by immunoprecipitation and immunoblot. **G** The methylation of endogenous RIP3 in HT29 cells treated with or without PRMT1 inhibitor TC-E 5003 (25 nM) for 6 h was analyzed by immunoprecipitation and immunoblot.

It has been well recognized that the asymmetric arginine di-methylation is catalyzed by type I arginine methyltransferases (PRMTs) including PRMT1, PRMT2, PRMT3, PMMT4, PRMT6 and PRMT8 [23, 25, 26], among them PRMT1 is the predominant enzyme [27]. To verify which PRMT is required for RIP3 methylation, we performed mass spectrum and PRMT1 was identified in the RIP3 interaction pull-down proteins (Fig. S2). We carried out the in vivo protein interaction assay of endogenous RIP3 with the asymmetric methyltransferases PRMT1, 2 and 4. PRMT1 but not PRMT2 or PRMT4 interacted with RIP3 (Fig. 2A). These data suggest that PRMT1 might be the potential methyltransferase of RIP3 protein.

We next examined the methyltransferase activity of PRMT1. Overexpression of PRMT1 promoted ADMA di-methylation and MMA mono-methylation of RIP3 but not RIP3^R486K^ mutant (Fig. 2B), while stable knockdown of PRMT1 eliminated RIP3 methylation in HT29 cells (Fig. 2C). We did in vitro methylation assay by incubating PRMT1 and RIP3 in the protein methylation assay buffer. The addition of PRMT1 increased ADMA and MMA forms of both commercial recombinant RIP3 and the purified RIP3 in our laboratory, but not RIP3^R486K^ protein (Fig. 2D, E), indicating that PRMT1 directly methylated RIP3 at R486. To further confirm the methyltransferase activity of PRMT1, we treated HT29 cells with two commercial PRMT1 inhibitors C-7280948 and TC-E 5003. Both of them reduced the levels of RIP3 methylation (Fig. 2F, G).

Collectively, we identify RIP3 as a new substrate of PRMT1 and PRMT1 mediates the mono-methylation and asymmetric di-methylation of human RIP3 at R486.

### Methylation inhibits RIP3 interaction with RIP1 to prevent necrosome complex

It has been well characterized that necroptosis activation requires the interaction of RIP3 with the upstream kinase RIP1 to form a necroptotic protein complex termed necrosome, where RIP1 phosphorylates and activates RIP3. The interaction of RIP3 and RIP1 kinase through the RHIM domains existing in both proteins is essential for the necroptosis activation. We have shown that RIP3 is methylated by PRMT1 at R486 and noticed that R486 amino acid locates at the proximity region of human RIP3 RHIM domain (Fig. 1G). We suspected that RIP3 methylation might affect the interaction of RIP3 with RIP1 to form RIP1-RIP3 complex, consequently regulating RIP3 phosphorylation and necroptosis activation.

**Fig. 3:**
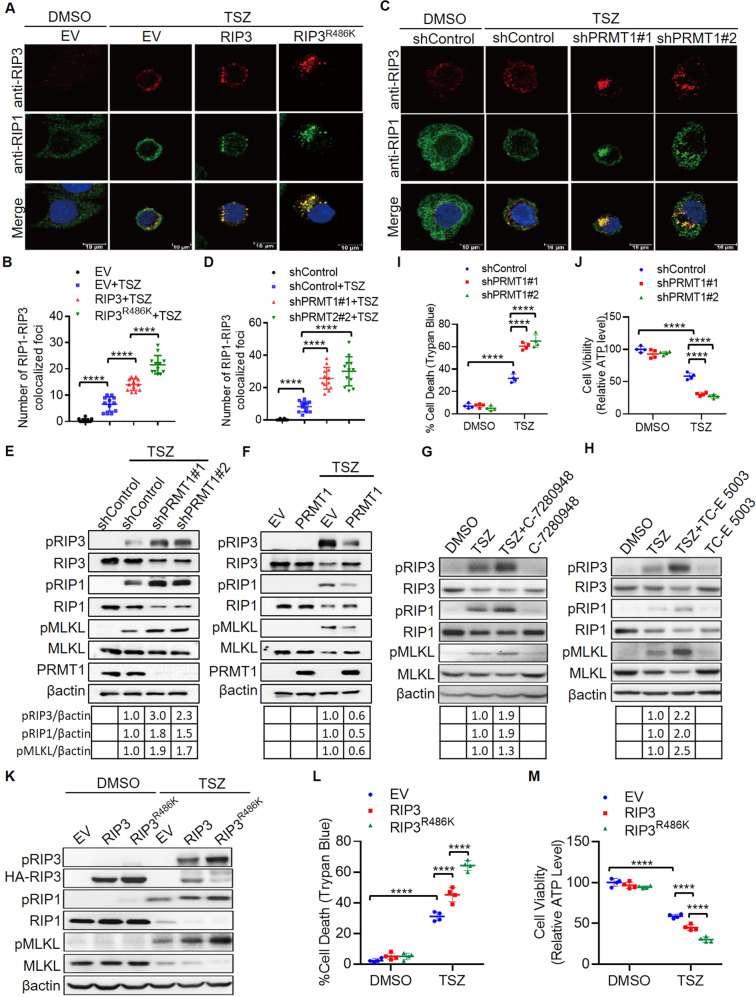
PRMT1-mediated RIP3 methylation inhibits RIP1-RIP3 complex formation to block necroptosis. **A** Immunofluorescence of RIP1-RIP3 colocalized complex foci in the control (EV), RIP3 and RIP3^R486^ stable overexpression HT29 cells treated with or without TSZ (20 ng/ml TNF-alpha, 100 nM LCL-161, 50 µM Z-VAD-fmk) for 6 h. **B** Statistical analysis of RIP1-RIP3 colocalized complex foci in the control (EV), RIP3 and RIP3^R486^ stable overexpression cells treated with or without TSZ for 6 h. *n* = 12. ****P < 0.0001. **C** Immunofluorescence of RIP1-RIP3 colocalized complex foci in the control and PRMT1 stable knockdown HT29 cells treated with or without TSZ for 6 h. **D** Statistical analysis of RIP1-RIP3 colocalized complex foci in the control and PRMT1 stable knockdown cells treated with or without TSZ for 6 h. *n* = 12. ****P < 0.0001. **E** The control and PRMT1 stable knockdown HT29 cells were treated with or without TSZ for 6 h. The indicated necroptosis proteins were analyzed by immunoblot. **F** The control (EV) and PRMT1 stable overexpression HT29 cells were treated with or without TSZ for 6 h. The indicated necroptosis proteins were analyzed by immunoblot. **G** HT29 cells were treated with or without TSZ and PRMT1 inhibitor C-7280948 (100 nM) for 6 h. The indicated necroptosis proteins were analyzed by immunoblot. **H** HT29 cells were treated with or without TSZ and PRMT1 inhibitor TC-E 5003 (25 nM) for 6 h. The indicated necroptosis proteins were analyzed by immunoblot. **I** The control and PRMT1 stable knockdown HT29 cells were treated with or without TSZ for 16 h. The percentage of dead cells was calculated by Trypan Blue staining. *n* = 4. ****P < 0.0001. **J** The control and PRMT1 stable knockdown HT29 cells were treated with or without TSZ for 16 h. Cell viability was analyzed by measuring cellular ATP level. *n* = 4. ****P < 0.0001. **K** The control (EV), RI*P*3 and RIP3^R486^ stable overexpression HT29 cells were treated with or without TSZ for 6 h. The indicated necroptosis proteins were analyzed by immunoblot. **L** The control (EV), RIP3 and RIP3^R486^ stable overexpression HT29 cells were treated with or without TSZ for 16 h. The percentage of dead cells was calculated by Trypan Blue staining. *n* = 4. ****P < 0.0001. **M** The control (EV), RIP3 and RI*P*3^R486^ stable overexpression HT29 cells were treated with or without TSZ for 16 h. Cell viability was analyzed by measuring cellular ATP level. *n* = 4. ****P < 0.0001.

To determine whether RIP3 methylation affects the interaction of RIP3 with RIP1. We examined the immunofluorescence foci of RIP1-RIP3 colocalized complex in the RIP3 and R486 mutant stable overexpression HT29 cells. Overexpression of RIP3 R486K mutant significantly increased the number of RIP1-RIP3 colocalized foci in comparison to that of wild-type RIP3 in the TSZ treated necrotic HT29 cells (Fig. 3A, B), suggesting that R486 methylation inhibits RIP3-RIP1 interaction and the necrosome formation. On the other hand, we also examined the role of PRMT1 in affecting RIP1-RIP3 complex. We found that the number of RIP1-RIP3 colocalized foci was significantly enhanced in PRMT1 deficient cells in which RIP3 methylation was absent (Fig. 3C, D).

Therefore, our data indicate that PRMT1-mediated R486 methylation inhibits the interaction of RIP3 and RIP1 to prevent necrosome protein complex formation, which might consequently lead to impaired RIP3 phosphorylation and necroptosis activation.

### PRMT1-mediated RIP3 methylation inhibits RIP3 phosphorylation and necroptosis

To investigate whether PRMT1-mediated RIP3 methylation regulates RIP3 and necroptosis activation, we treated PRMT1 control and stable knockdown HT29 cells with TSZ to induce necroptosis. PRMT1 knockdown cells displayed the enhanced phosphorylation of RIP3, as well as the higher levels of pRIP1 and pMLKL (Fig. 3E). In contrast, PRMT1 overexpression reduced the phosphorylation of RIP3, RIP1 and MLKL upon TSZ treatment (Fig. 3F). To further verify the role of PRMT1, we treated the cells with PRMT1 inhibitors C-7280948 and TC-E 5003 respectively, both C-7280948 and TC-E 5003 increased the levels of pRIP3, pRIP1 and pMLKL(Fig. 3G, H) and the necroptotic morphology (Fig. S3A) in the TSZ-induced necroptotic cells. It is known that the activation of necroptosis signaling leads to necroptotic cell death. We analyzed the cell death and viability by counting the Trypan Blue positive death cells and measuring the ATP level of survival cells. Notably, the silencing of PRMT1 significantly increased the necrotic cell death and decreased the viability of TSZ treated cells (Figs. 3I, J and S3B).

We next verified the role of R486 methylation in necroptosis. We established the wild-type RIP3 and mutant RIP3^R486K^ stable overexpression HT29 cell lines. TSZ promoted the phosphorylation of overexpressed RIP3 as well as endogenous RIP1 and MLKL. In the RIP3^R486K^ overexpression cells, the levels of RIP3, RIP1 and MLKL phosphorylation were higher than that in the wild-type RIP3 overexpression cells (Fig. 3K). Consistently, TSZ treatment led to more cell death but less cell viability in the mutant RIP3^R486K^ overexpression cells than that in the wild-type RIP3 overexpression cells (Figs. 3L, M and S3C), confirming that RIP3 methylation at R486 negatively regulates RIP3 activation and necroptosis.

Our data demonstrate that PRMT1-mediated RIP3 R486 methylation inhibits necroptosis through suppressing RIP1-RIP3 necrosome complex formation.

### Lack of RIP3 methylation promotes colon cancer immune escape via MDSC

**Fig. 4:**
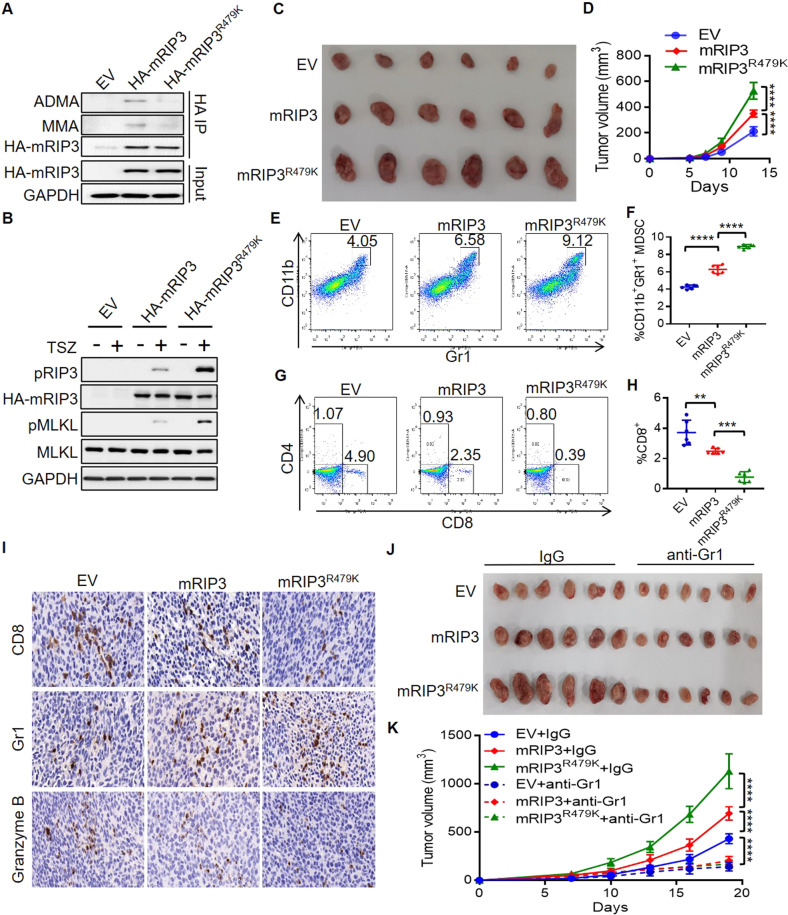
Lack of RIP3 methylation enhances colon cancer growth through MDSC. **A** The methylation of murine RIP3 (mRIP3) and mRIP3^R479K^ mutant was analyzed by immunoprecipitation and immunoblot in stable overexpression CT26 cells. **B** The control (EV), mRIP3 and mRIP3^R479^ stable overexpression CT26 cells were treated with or without TSZ for 6 h. The indicated necroptosis proteins were analyzed by immunoblot. **C** The images of the control (EV), mRIP3, mRIP3^R479K^ stable overexpression CT26 tumors. **D** The tumor growth curve of the control (EV), mRIP3, mRIP3^R479K^ stable overexpression CT26 tumors. *n* = 6. ****P < 0.0001. **E** Representative flowcytometry analysis of CD11b^+^Gr1^+^ MDSC in the control (EV), mRIP3, mRIP3^R479K^ stable overexpression CT26 tumors. **F** Flowcytometry statistical analysis of CD11b^+^Gr1^+^ MDSC percentage in the control (EV), mRIP3, mRIP3^R479K^ stable overexpression CT26 tumors. *n* = 6. ****P < 0.0001. **G** Representative flowcytometry analysis of CD8^+^ and CD4^+^ T cells in the control (EV), mRIP3, mRIP3^R479K^ stable overexpression CT26 tumors. **H** Flowcytometry statistical analysis of CD8^+^ T cell percentage in the control (EV), mRIP3, mRIP3^R479K^ stable overexpression CT26 tumors. *n* = 6. **P < 0.01, ***P < 0.001. **I** Immunohistochemistry staining of CD8^+^, Gr1^+^ and Granzyme B^+^ cells in the control (EV), mRIP3, mRIP3^R479K^ stable overexpression CT26 tumors. **J** The images of the control (EV), mRIP3, mRIP3^R479K^ stable overexpression CT26 tumors treated with or without anti-Gr1 antibody. **K** Th tumor growth curve of the control (EV), mRIP3, mRIP3^R479K^ stable overexpression CT26 tumors treated with or without anti-Gr1 antibody. *n* = 6. ****P < 0.0001.

Despite that necroptosis lead to cell death, RIP3-dependent necroptosis promotes cancer progression through suppressing anti-tumor immunity, particularly via recruiting MDSC cells that subsequently inhibiting T cell activation [28, 29]. In order to explore the role of RIP3 methylation in tumor immunity, we investigated the effects of murine wild-type RIP3 and RIP3 methylation deficient mutant on the progression of murine CT26 colon cancer in immunocompetent Balb/c mice. Sequence alignment revealed that murine RIP3 arginine 479 was the homologous amino acid of arginine 486 in human RIP3 (Fig. S4A). We generated murine wild-type RIP3 (mRIP3) and R479K mutant (mRIP3^R479K^) stable overexpression CT26 cells and found that mRIP3 was methylated but mRIP3^R479K^ was not, confirming that R479 is the conserved methylation arginine in murine RIP3 (Fig. 4A). In addition, the necroptosis signaling transduction was also enhanced in mRIP3^R479K^ cells in comparison to that in wild-type mRIP3 cells (Fig. 4B). Therefore, mRIP3 arginine 479 methylation inhibits necroptosis the same as that of homologous human RIP3 R486 methylation.

We then established mRIP3 and mRIP3^R479K^ stable overexpression subcutaneous CT26 colon tumor in Balb/c mice. mRIP3 promoted tumor growth, while mRIP3^R479K^ CT26 tumors grew faster than mRIP3 tumors (Fig. 4C, D). Flowcytometry analysis of the tumor infiltrating immune cells showed that mRIP3 overexpression enhanced tumor immune suppressive CD11b^+^Gr1^+^MDSC cells and reduced CD8^+^ T cells, but the ratio of CD4^+^ T cells remained unaltered (Figs. 4E–H and S4B). mRIP3^R479K^ overexpression further increased the ratio of MDSC but decreased the ratio of CD8^+^ T cells compared to that of mRIP3 overexpression (Fig. 4E–H). Consistently, immunohistochemistry staining confirmed that mRIP3^R479K^ overexpression increased tumor Gr1^+^ MDSC, while inhibited CD8^+^ T cells and Granzyme B^+^ cytotoxic cells (Figs. 4I and S4C–E). Anti-Gr1 antibody (RB6-8C5) has been shown to inhibit MDSC and revert tumor immune evasion [30, 31]. When the mice were treated with anti-Gr1 antibody (RB6-8C5) (Fig. S4F), the tumor promoting effects of either mRIP3 or mRIP3^R479K^ were restored to the level of empty vector controls (Fig. 4J, K).

Thus, lack of RIP3 methylation builds an immune evasion microenvironment contributing to tumor progression mainly via MDSC immune suppressor cells.

### PRMT1 reverts the immune escape of RIP3 necroptotic colon cancer

**Fig. 5:**
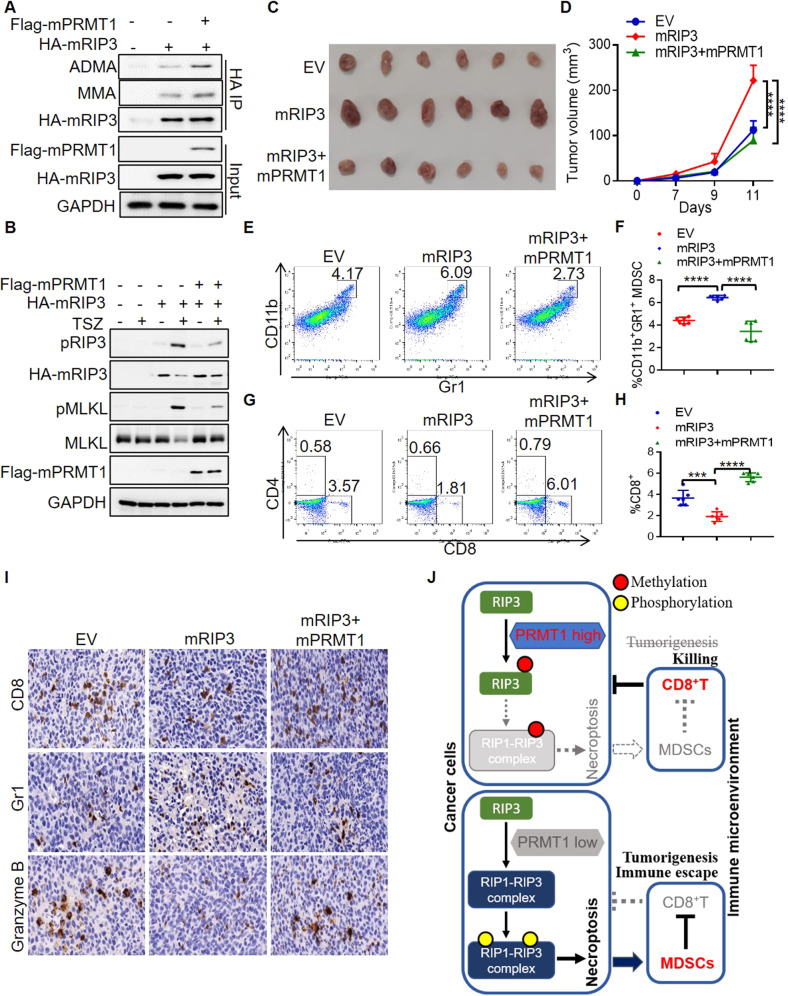
PRMT1 inhibits RIP3 to prevent colon cancer immune escape. **A** The methylation of murine RIP3 (mRIP3) in CT26 cells with or without murine PRMT1 (mPRMT1) overexpression was analyzed by immunoblot. **B** The control (EV), mRIP3 and mRIP3 + mPRMT1 stable overexpression CT26 cells were treated with or without TSZ for 6 h. The indicated necroptosis proteins were analyzed by immunoblot. **C** The images of the control (EV), mRIP3, mRIP3 + mPRMT1 stable overexpression CT26 tumors. **D** Th tumor growth curve of the control (EV), mRIP3, mRIP3 + mPRMT1 stable overexpression CT26 tumors. *n* = 6. ****P < 0.0001. **E** Representative flowcytometry analysis of CD11b^+^Gr1^+^ MDSC in the control (EV), mRIP3, mRIP3 + mPRMT1 stable overexpression CT26 tumors. **F** Flowcytometry statistical analysis of CD11b^+^Gr1^+^ MDSC percentage in the control (EV), mRIP3, mRIP3 + mPRMT1 stable overexpression CT26 tumors. *n* = 6. ****P < 0.0001. **G** Representative flowcytometry analysis of CD8^+^ and CD4^+^ T cells in the control (EV), mRIP3, mRIP3 + mPRMT1 stable overexpression CT26 tumors. **H** Flowcytometry statistical analysis of CD8^+^ T cell percentage in control (EV), mRIP3, mRIP3 + mPRMT1 stable overexpression CT26 tumors. *n* = 6. ***P < 0.001, ****P < 0.0001. **I** Immunohistochemistry staining of CD8^+^, Gr1^+^ and Granzyme B^+^ cells in the control (EV), mRIP3, mRIP3 + mPRMT1 stable overexpression CT26 tumors. **J** Schematic model. In PRMT1 high cells, PRMT1 methylates RIP3 to inhibit RIP1-RIP3 necrosome complex formation and necroptosis, evoking anti-tumor immune responses. In PRMT1 low cells, RIP3 interacts with RIP1 to form RIP1-RIP3 necrosome complex, leading to RIP3 phosphorylation and necroptosis activation that promotes the colon cancer immune escape through MDSC accumulation and CD8^+^ T inhibition.

As we have shown that human PRMT1 serves as a negative regulator of necroptosis in human colon cancer by methylating RIP3, we are interested in knowing whether murine PRMT1 (mPRMT1) is consistently involved in RIP3 methylation and necroptosis inhibition. To this end, we constructed murine PRMT1 (mPRMT1) and generated mRIP3 and mPRMT1 double overexpression CT26 murine cells. Indeed, mPRMT1 methylated mRIP3 (Fig. 5A). TSZ induced RIP3 phosphorylation in the mRIP3 stable overexpression CT26 cells, while double overexpression with mPRMT1 abolished the effect of mRIP3 phosphorylation thus suppressing necroptosis (Fig. 5B). Taking together, murine PRMT1 methylates RIP3 to inhibit RIP3-dependent necroptosis the same as that of human homologs.

We then examined whether murine PRMT1 is also involved in the colon cancer immunity as a mRIP3 methyltransferase. We found that mRIP3 promoted CT26 colon cancer growth, while the promoting effect of mRIP3 was reverted by mPRMT1(Fig. 5C, D), suggesting that mPRMT1 inhibits the mRIP3-dependent colon cancer growth. In addition, flowcytometry analysis of tumor infiltrating immune cells showed that mPRMT1 restored the increased immune suppressive CD11b^+^Gr1^+^MDSC and reduced CD8^+^ T cells of mRIP3 tumors, but not affecting the ratio of CD4^+^ T cells (Figs. 5E–H and S5A). Immunohistochemistry staining further confirmed that mPRMT1 reversed the increased infiltration of Gr1^+^ MDSC and decreased CD8^+^ T cells and Granzyme B^+^ cytotoxic cells that resulted from mRIP3 (Figs. 5I and S5B–D).

Collectively, our findings indicate that PRMT1 serves as an arginine methyltransferase of RIP3 in both human and mouse species. PRMT1 methylates RIP3 to inhibit RIP1-RIP3 complex formation, resulting in the impaired RIP3 phosphorylation and necroptosis activation that builds the beneficial immune microenvironment against colon cancer growth (Fig. 5J).

### PRMT1 and RIP3 R486^ADMA^ predicts the prolonged colon cancer patient survival

**Fig. 6:**
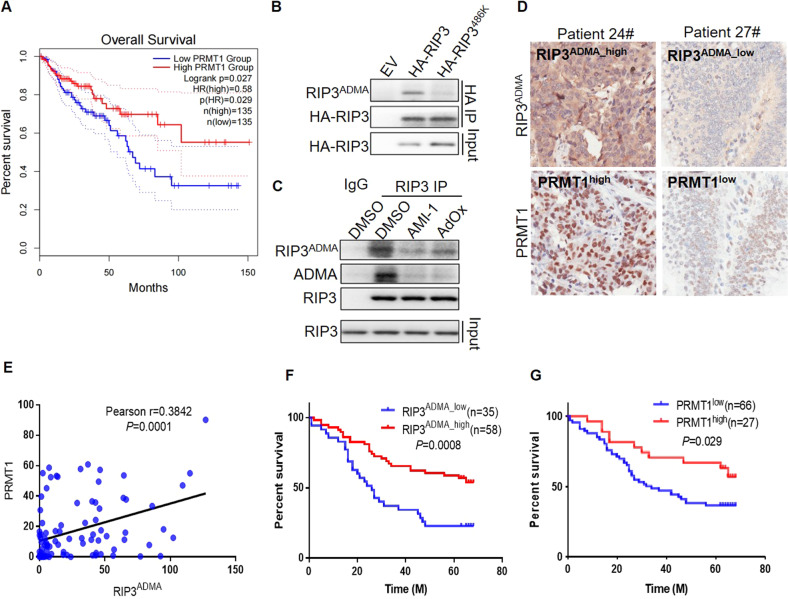
PRMT1 and RIP3 R486^ADMA^ predict the prolonged colon cancer patient survival. **A** GEPIA overall survival analysis of colon carcinoma patients with low and high PRMT1 mRNA expression in TCGA database. **B** The methylation of overexpressed RIP3 and RIP3^486K^ was analyzed with the R486 methylation specific antibody RIP3^ADMA^ in stable overexpression HT29 cells. **C** The methylation of endogenous RIP3 in HT29 cells treated with or without AMI-1 and AdOx was analyzed with the R486 methylation specific antibody RIP3^ADMA^. **D** Representative immunohistochemistry staining of human colon cancer tissue sections with PRMT1 and RIP3^ADMA^. **E** The positive correlation of PRMT1 protein with RIP3^ADMA^ methylation levels in human colon cancer samples. Pearson r = 0.3842. *P* = 0.0001. **F** Overall survival analysis of colon cancer patients with low and high RIP3^ADMA^ expression. *P* = 0.0008. **G** Overall survival analysis of colon cancer patients with low and high PRMT1 protein expression. *P* = 0.029.

We have shown that PRMT1 inhibits the colon cancer immune escape and tumor growth via RIP3 methylation in the mouse colon cancer. We would like to investigate the pathological significance of PRMT1-RIP3 axis in human colon cancer patients. By utilizing GEPIA (gepia.cancer-pku.cn) web tools, we analyzed the TCGA colon adenocarcinoma datasets. Though the mRNA level of PRMT1 in colon cancer tissues tended to be higher than that in cancer adjacent tissues (Fig. S6A), the high PRMT1 expression in the colon cancer tissues was associated with the longer patient survival (Fig. 6A), supporting a role of PRMT1 in preventing the tumor malignancy. Simultaneously, we investigated RIP3 mRNA expression and found that it was lower in cancer tissues than that in adjacent normal tissues (Fig. S6B). In addition, TCGA database showed that the high RIP3 mRNA expression in cancer was associated with the prolonged patient survival (Fig. S6C). However, multiple studies have reported that RIP3 promotes the colon cancer progression [28, 32]. Our experimental data also demonstrated that RIP3 protein, particularly the RIP3 mutant lack of arginine methylation favored the tumor immune evasion microenvironment to promote colon cancer growth. Thus, it is essential to evaluate the levels of RIP3 methylation but not only the mRNA level of RIP3 in the patient samples.

As RIP3 was methylated by the asymmetric methyltransferase PRMT1, we generated an RIP3^ADMA^ antibody to specifically detect the asymmetric di-methylated R486 of RIP3. Dot blot assay verified the specificity of RIP3^ADMA^ antibody that recognizing the peptide containing asymmetric di-methylated R486 (GKGR^ADMA^GLQ-C) but not the control peptide without modification (GKGRGLQ-C) (Fig. S6D). By using this RIP3^ADMA^ specific antibody, we confirmed that wild-type RIP3 but not R486K mutant was methylated (Fig. 6B). RIP3^ADMA^ antibody also detected the methylated form of endogenous RIP3 in HT29 cells (Fig. 6C). The signal recognized by RIP3^ADMA^ antibody was consistent with that of pan ADMA antibody and was abolished by the methyltransferase inhibitors AdOx and AMI-1 (Fig. 6C). Therefore, we successfully generated the R486 methylation specific antibody for measuring RIP3 methylation in human samples.

With the RIP3^ADMA^ antibody, we were able to characterize the protein levels of RIP3 R486 methylation together with PRMT1 in the colon adenocarcinoma tissue chip containing the colon cancer samples from 93 patients (Table S1). Consistent with the notion that PRMT1 methylates RIP3, the protein level of RPMT1 was significant positively correlated with RIP3^ADMA^ level in the patient samples (Fig. 6D, E). Importantly, the patients with RIP3^ADMA^ high expression cancer survived longer, while low RIP3^ADMA^ patients showed poor survival (Fig. 6F). Consistently, the high expression of PRMT1 protein was associated with the long overall survival of colon cancer patients (Fig. 6G). Thus, the protein levels of either RIP3^ADMA^ or PRMT1 are beneficial indicators of colon cancer patients.

Clinical analysis not only confirms the tumor suppressive role of RIP3 methylation and PRMT1, but also suggests that RIP3^ADMA^ and PRMT1 can be the molecular markers for evaluating the colon adenocarcinoma malignancy.

## Discussion

Cancer develops strategies to counter against the destiny of cell death, a hallmark of cancer cells. Forcing cancer cell death is the principle strategy for treating the disease [33]. As known for necroptosis resistance, certain types of cancer cells express low level or even absence of RIP3 due to DNA hypermethylation. The induction of necroptotic cell death through restoring RIP3 expression has been shown to facilitate cancer chemotherapies [1, 34]. In contrast, though RIP3 is silenced in some cancer cell lines, many other cancer cells including the colon cancer cell HT29 display relatively high level of RIP3 [1, 22]. It is unclear whether and how necroptosis can be concisely controlled in the high RIP3 expression cancer. With the colon cancer models, we identify that RIP3 protein is methylated at R486 of human RIP3 and the conserved amino acid R479 in the murine homolog. RIP3 protein methylation serves a break of RIP3 phosphorylation and necroptosis activation. Thus, in addition to DNA methylation, the protein methylation of RIP3 may also be utilized to inhibit necroptosis.

Despite that necroptosis can lead to cancer cell death, the role of necroptosis in tumorigenesis is dependent on the context of cancer in vivo [4]. It is believed that chronic inflammation caused by the release of DAMPs from necrotic cells can promote tumor progression. RIP3 is upregulated in the human pancreatic ductal adenocarcinoma (PDA). Genetic RIP3 knockout in mouse inhibits the progression of PDA by remodeling immune microenvironment [29]. In the mouse colon cancer models induced by azoxymethane (AOM) and dextran sodium sulfate (DSS), RIP3 level also upregulates in the tumor tissues. The deletion of RIP3 suppresses colon tumorigenesis, which is associated with reduced immune suppressive MDSC cells as well as increased T cells [32]. In addition, mTOR has been found to inhibit RIP3 degradation, resulting in enhanced necroptosis, colon inflammation and tumorigenesis [17]. Parkin ubiquitinates RIP3 to prevent RIP1-RIP3 necrosome formation, RIP3 phosphorylation and necroptosis thereby blocking inflammation and tumorigenesis [18]. Another study further show that the inhibition of RIP3 kinase activity by a small molecular GSK872 reduces the infiltration of intermediate MDSC to suppress intestinal tumorigenesis [28]. Our findings in this study confirm that RIP3 promotes colon cancer growth through MDSC. Importantly, we demonstrate that the methylation of RIP3 protein serves as a break of necroptosis, immune escape and colon cancer progression in vivo.

PRMT1 is a dominant member of the asymmetric arginine methyltransferase family [27]. The expression of PRMT1 is associated with multiple cancers including colon cancer [23]. To date, though PRMT1 has been usually recognized as an oncogenic protein [35, 36], emerging evidence has occurred that PRMT1 may negatively regulate proliferation and invasion [37]. Importantly, it has been reported that PRMT1 displays the tumor suppressive function by methylating p14^ARF^ to promote apoptosis upon genotoxic stress. High levels of PRMT1 are associated with beneficial prognosis of pancreatic ductal adenocarcinoma patients [38]. Interestingly, the patient survival analysis of TCGA datasets in our study reveals that PRMT1 mRNA level is also positively associated with colon cancer patient life span. In addition to mRNA level, we show that colon cancer PRMT1 protein is consistently higher in the patients who survive longer. Furthermore, we demonstrate that PRMT1 inhibits the necroptosis by methylating RIP3, leading to the anti-tumor immune responses and colon cancer suppression. Thus, PRMT1 can be a tumor suppressor in the necroptotic colon cancer.

Our study demonstrate that PRMT1 suppresses colon cancer, which can provide a more comprehensive knowledge of the underlying mechanisms of PRMT1 in cancer progression. PRMT1 has been known as an oncogene via stimulating cancer cell proliferation or blocking apoptosis [36, 39], while the opposite findings have also been reported in some experiments in which PRMT1 might inhibit proliferation and promote apoptosis in cancer [37, 38]. Despite of regulating cancer cell proliferation and apoptosis, we provide another mechanism driven by PRMT1, in which PRMT1 inhibits necroptotic cell death. It is well known that the necroptosis causes cancer cell death on one side, but on the other side, necroptotic cell death can induce cancer inflammation through MDSC and promote immune escape, particular in the cancer types closely related to chronic inflammation, such as colon cancer [32]. Thus, the known oncogenic functions of PRMT1 are mainly regulated through cancer cell proliferation or apoptosis, while the tumor suppressor function of PRMT1 in colon cancer is likely via the suppression of MDSC inflammation microenvironment by inhibiting necroptosis.

Notably, PRMT1 methylates RIP3 to inhibit necroptotic cell death while compromising necroptosis-dependent immune resistant microenvironment. The induction of necroptosis by targeting PRMT1 will not only cause cancer cell death, but also increase the immune escape of colon cancer cells. Due to the double-edged sword role of PRMT1, we may propose a potential therapeutic strategy to target PRMT1 together with MDSC and T cell immune checkpoint.

In summary, our study reveals a molecular mechanism of necroptosis and colon cancer immunity that is regulated by PRMT1. PRMT1 inhibits RIP3 phosphorylation and necroptosis activation by methylating RIP3 protein, thereby preventing the necroptosis-dependent immune escape and evoking the beneficial immune microenvironment. In addition, we show that the protein levels of PRMT1 and RIP3 methylation serve as the molecular markers of colon cancer prognosis.

## Materials and methods

### Cell culture and reagents

Human HEK293T cells, human HT29 colon cancer cells and murine CT6 colon cancer cells were used in this study and cultured in high glucose DMEM supplied with 10% FBS. TNF-alpha (R&D, 210-TA-020), LCL-161 (Fisher, NC0605628), Z-VAD-fmk (Fisher, FMK001), Z-VAD-fmk (Selleck, S7023), AdOx (Sigma, A7154), AMI-1 (Selleck, S7884), C-7280948 (Selleck, S6737) and TC-E 5003 (Selleck, S0855) reagents were used to treat cells with the dosage and time indicated in figure legend.

### Immunoblot, immunoprecipitation and immunofluorescence

Briefly, the cells were washed with cold PBS and lysed in RIPA buffer containing protease inhibitor cocktail (Roche) and phosphatase inhibitors (10 mM Na-pyrophosphate,10 mM Na-glycerophosphate, 50 mM Na-fluoride)) followed by SDS electrophoresis and immunoblot of indicated proteins. For immunoprecipitation, the lysates with equal total proteins were incubated with primary antibody overnight at 4 °C, followed by incubation with protein agarose A/G beads to pulldown antibody specific proteins, the beads were then washed with lysis buffer and subjected for immunoblot analysis. The antibodies used for immunoblot and immunoprecipitation included RIP1 (D94C12) (CST, 3493), Phospho-RIP1 (Ser166) (D8I3A) (CST, 44590), RIP3 (E1Z1D) (CST, 13526), RIP3 (SCBT, sc-374639), Phospho-RIP3 (Ser227) (D6W2T) (93654), MLKL(3H1) (Millipore, MABC604), Phospho-MLKL (EPR9514) (Abcam, ab187091), Phospho-MLKL (D6H3V) (CST, 91689)Rodent Phospho-RIP3 (Thr231/Ser232) (E7S1R) (CST, 91702), Rodent RIP3 (D4G2A) (CST, 95702), Rodent Phospho-RIP (Ser166) (E7G6O) (CST, 31122), Rodent Phospho-MLKL (Ser345) (D6E3G) (CST, 37333), Rodent MLKL (D6W1K) (CST, 37705), HA (16B12) (Biolegend, 901515), FLAG M2 (Sigma, F1804), Mono-Methyl Arginine [mme-R] (CST, 8015), Asymmetric Di-Methyl Arginine Motif [adme-R] (CST, 13522), Symmetric Di-Methyl Arginine Motif [sdme-RG] (CST, 13222), RIP1 (C-12) (Santa cruz, sc-133102), RIP3 (B-2) (Santa cruz, sc-374639), PRMT1 (B-2) (Santa cruz, 166963), PRMT2 (B-11) (Santa cruz, sc-393254), PRMT4 (D6) (Santa cruz, sc-390656), β-Actin (AC 15) (Sigma, A5441). For immunofluorescence, the cells cultured in Nunc™ Lab-Tek™ Chamber Slide were fixed with 4% paraformaldehyde for 10 min, permeabilized with 0.3%Triton for 10 min and blocked with 2% BSA for 10 min at room temperature, followed by incubation with RIP1 (D94C12) (CST, 3493) and RIP3 (Santa Cruz, sc-374639) primary antibodies overnight at 4 °C and fluorescent second antibody at room temperature for 1 h. The slides were then co-mounted with DAPI and the confocal images were taken. RIP1/RIP3 co-localized foci were counted per cell. *n* = 12 cells from per group were statistically analyzed. Two biologically independent replicates were performed. Uncropped western blots were shown in Supplemental Material.

### In vitro methylation assay

For in vitro methylation assay, 0.5 μg purified methyltransferases PRMT1, PRMT2 or PRMT4 were incubated with 0.5 μg recombinant RIP3 protein or purified RIP3 and RIP3^R486K^ mutant proteins in 30 μl methylation reaction buffer of 1xPBS with 1mM S-adenosyl-L-methylmethionine for 90 min at 30 °C. The methylation of RIP3 was measured following RIP3 pulldown and immunoblot.

### Animal study

6–8 weeks age female Balb/c immunocompetent mice were used for tumor xenograft experiments. The tumor xenografts were established by subcutaneously injecting 1 million murine CT26 cells in Balb/c mice. For anti-Gr1 antibody (RB6–8C5) treatment, the mice were injected intraperitoneally with IGG or anti-Gr1 antibody 250 μg per mouse every 3 days from 1 day after tumor inoculation. *n* = 6 mice per group. The sample sizes were chosen for the requirement of statistical analysis. The mice were randomly divided into different groups. Investigators were blinded to the group allocation. The tumor diameters (width and length) were measured by a caliper and the volume were calculated as width x width x length/2. At the end of the experiments, the tumor tissues were dissected, digested with collagenase IV and Dnase I to prepare cell suspension for flowcytometry analysis of MDSC and T cells. The flowcytometry antibodies included CD11b (M1/70, Biolegend), Gr1 (RB6-8C5, Biolegend), CD4 (Biolegend), CD8a (Biolegend). Immunohistochemistry staining of the tumor tissue sections was performed with the antibodies of CD8a (D4W2Z) (CST, 98941), Gr1/Ly-6G (E6Z1T) (CST, 87048), Granzyme B (D6E9W) (CST, 46890). Infiltrating immune cells were quantified as the percentage of antibody staining positive cells to total cells in each taken picture. *n* = 3 images were statistically analyzed. Two biologically independent replicates were performed. All animal study followed the approved ethic procedures by the responsible committees in Guangdong Provincial People’s Hospital.

### Human patient samples

93 colon cancer samples on a tissue chip (HColA180Su12, Shanghai OUTDO Biotech) were used for PRMT1 (B-2) (Santa cruz, 166963) and RIP3^ADMA^ immunohistochemistry staining. The immunostaining signal density of PRMT1 and RIP3^ADMA^ was measured by image-J software and IHC score was quantified as integrated optical density (IOD) value/area. The patient sample study and the informed consent were in compliance with all relevant ethical regulations approved by Shanghai OUTDO Biotech committees and Guangdong Provincial People’s Hospital.

### Statistical analysis and reproducibility

Statistical analysis was performed with Graphpad Prism software. Log-rank (Mantel Cox) test was used for patient survival analysis. One-way ANOVA was used for analyzing the data with three or more groups. Pearson correlation coefficient analysis was used to analyze the correlation of PRMT1 protein with RIP3^ADMA^ methylation levels in human colon cancer samples. The samples sizes were chosen for the requirement of statistical analysis. No samples were excluded from the analysis. Statistical data was presented as mean ± SD. *P* < 0.05 represents statistical significance. * indicates *P* < 0.05; ** indicates *P* < 0.01; *** indicates *P* < 0.001;**** indicates *P* < 0.0001.

## Supplementary information

Supplemental figures and tables

Supplemental material_uncropped figures

Checklist

## Data Availability

All data supporting this study are available from the corresponding authors on reasonable request.
