# Supplementary material for: PRMT1 reverts the immune escape of necroptotic colon cancer through RIP3 methylation
Source: Cell Death Dis. 2023 Apr 1;14(4):233. doi: 10.1038/s41419-023-05752-w (PMC10067857; doi:10.1038/s41419-023-05752-w)
Supplement: Supplementary file 1 — Supplemental figures and tables [file 41419_2023_5752_MOESM1_ESM.pdf]

Supplementary Information for

PRMT1 reverts the immune escape of necroptotic colon cancer through RIP3 methylation

Lian Zhang[#], Yujiao He[#], Yi Jiang, Qi Wu, Yanchen Liu, Qingqiang Xie, Yuxiu Zou, Jiaqian Wu, Chundong Zhang, Zhongjun Zhou[*], Xiu-Wu Bian[*], Guoxiang Jin[*]

*Corresponding author. Email: gxjinking@163.com; bianxiuwu@263.net; zhongjun@hku.hk

This PDF file includes:

Figures S1 to S6
Tables S1

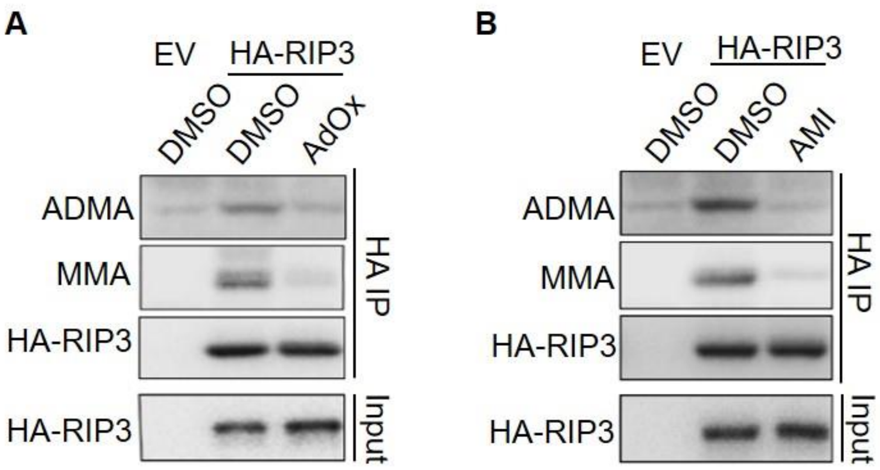

Figure S1 RIP3 undergoes arginine methylation. (**A**) The methylation of overexpressed RIP3 in HEK293T cells treated with or without AdOx was analyzed by immunoprecipitation and immunoblot. (**B**) The methylation of overexpressed RIP3 in HEK293T cells treated with or without AMI-1 was analyzed by immunoprecipitation and immunoblot.

HA-RIP3 IP Mass Spectrum

sp	P07437	TBB5_HUMAN	Tubulin beta chain OS=Homo sapiens OX=9606 GN=TUBB PE=1 SV=2	
sp	Q9Y572	RIPK3_HUMAN	Receptor-interacting serine/threonine-protein kinase 3 OS=Homo sapiens OX=9606 GN=RIPK3 PE=1 SV=2	RIP3
sp	Q71U36	TBA1A_HUMAN	Tubulin alpha-1A chain OS=Homo sapiens OX=9606 GN=TUBA1A PE=1 SV=1	
sp	P63261	ACTG_HUMAN	Actin, cytoplasmic 2 OS=Homo sapiens OX=9606 GN=ACTG1 PE=1 SV=1	
sp	Q96S66	CLCC1_HUMAN	Chloride channel CLIC-like protein 1 OS=Homo sapiens OX=9606 GN=CLCC1 PE=1 SV=1	
sp	P09211	GSTP1_HUMAN	Glutathione S-transferase P OS=Homo sapiens OX=9606 GN=GSTP1 PE=1 SV=2	
sp	P08670	VIME_HUMAN	Vimentin OS=Homo sapiens OX=9606 GN=VIM PE=1 SV=4	
sp	Q99873	ANM1_HUMAN	Protein arginine N-methyltransferase 1 OS=Homo sapiens OX=9606 GN=PRMT1 PE=1 SV=3	PRMT1
sp	Q9BY77	PDIP3_HUMAN	Polymerase delta-interacting protein 3 OS=Homo sapiens OX=9606 GN=POLDIP3 PE=1 SV=2	
sp	P61247	RS3A_HUMAN	40S ribosomal protein S3a OS=Homo sapiens OX=9606 GN=RPS3A PE=1 SV=2	
sp	P31943	HNRH1_HUMAN	Heterogeneous nuclear ribonucleoprotein H OS=Homo sapiens OX=9606 GN=HNRNPH1 PE=1 SV=4	
sp	Q53GQ0	DHB12_HUMAN	Very-long-chain 3-oxoacyl-CoA reductase OS=Homo sapiens OX=9606 GN=HSD17B12 PE=1 SV=2	

Figure S2 PRMT1 interacts with RIP3. Mass spectrum identified PRMT1 as a RIP3 interaction protein.

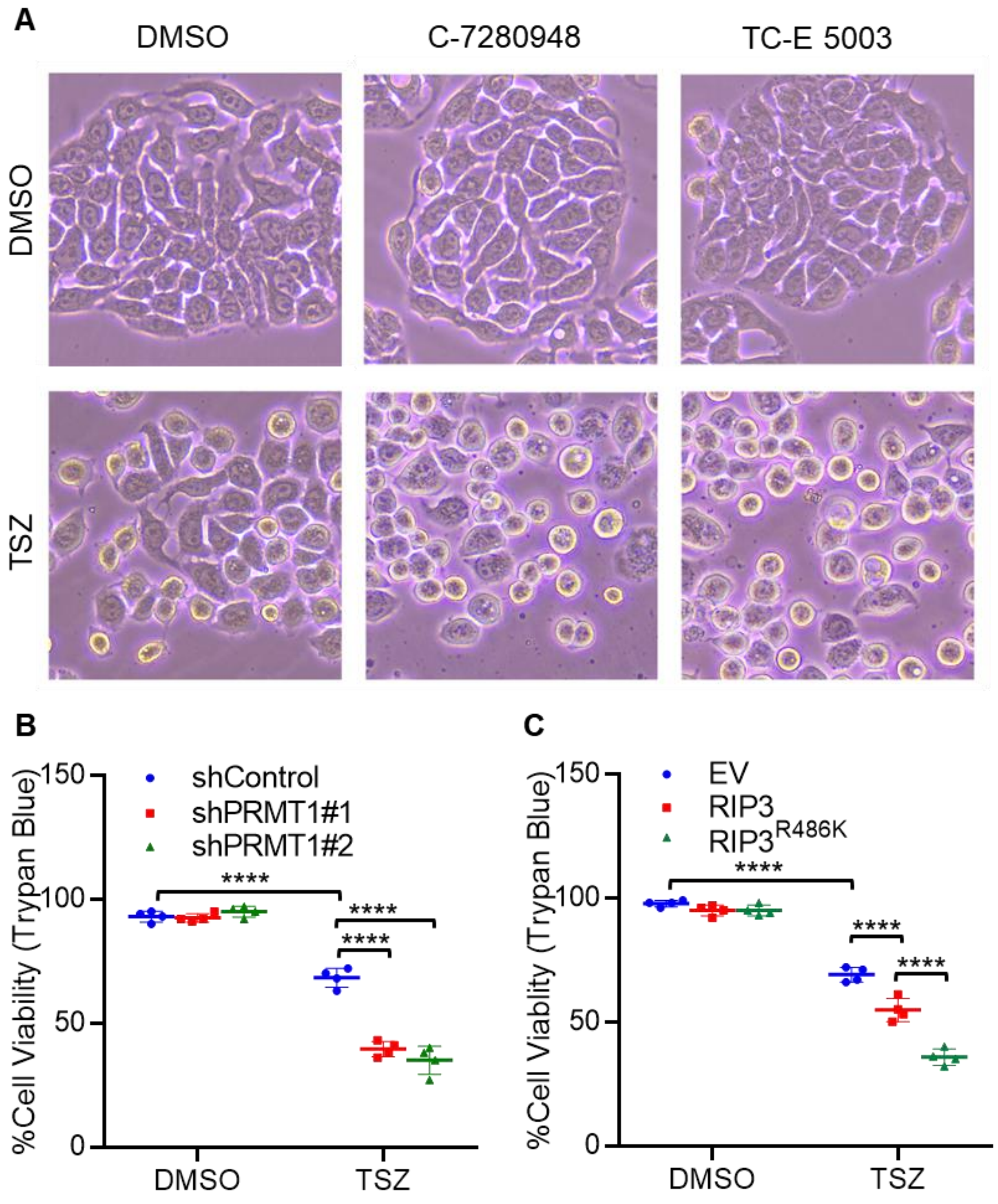

Figure S3 PRMT1 inhibits necroptosis. (A) The morphology of HT29 cells treated with or without TSZ and PRMT1 inhibitors C-7280948 (100nM), TC-E 5003 (25nM) for 8 hours. (**B**) The control and PRMT1 stable knockdown HT29 cells were treated with or without TSZ for 16 hours. Cell viability was calculated by Trypan Blue staining. n=4. ****P<0.0001. (**C**) The control (EV), RIP3 and RIP3^{R486} stable overexpression HT29 cells were treated with or without TSZ for 16 hours. Cell viability was calculated by Trypan Blue staining. n=4. ****P<0.0001.

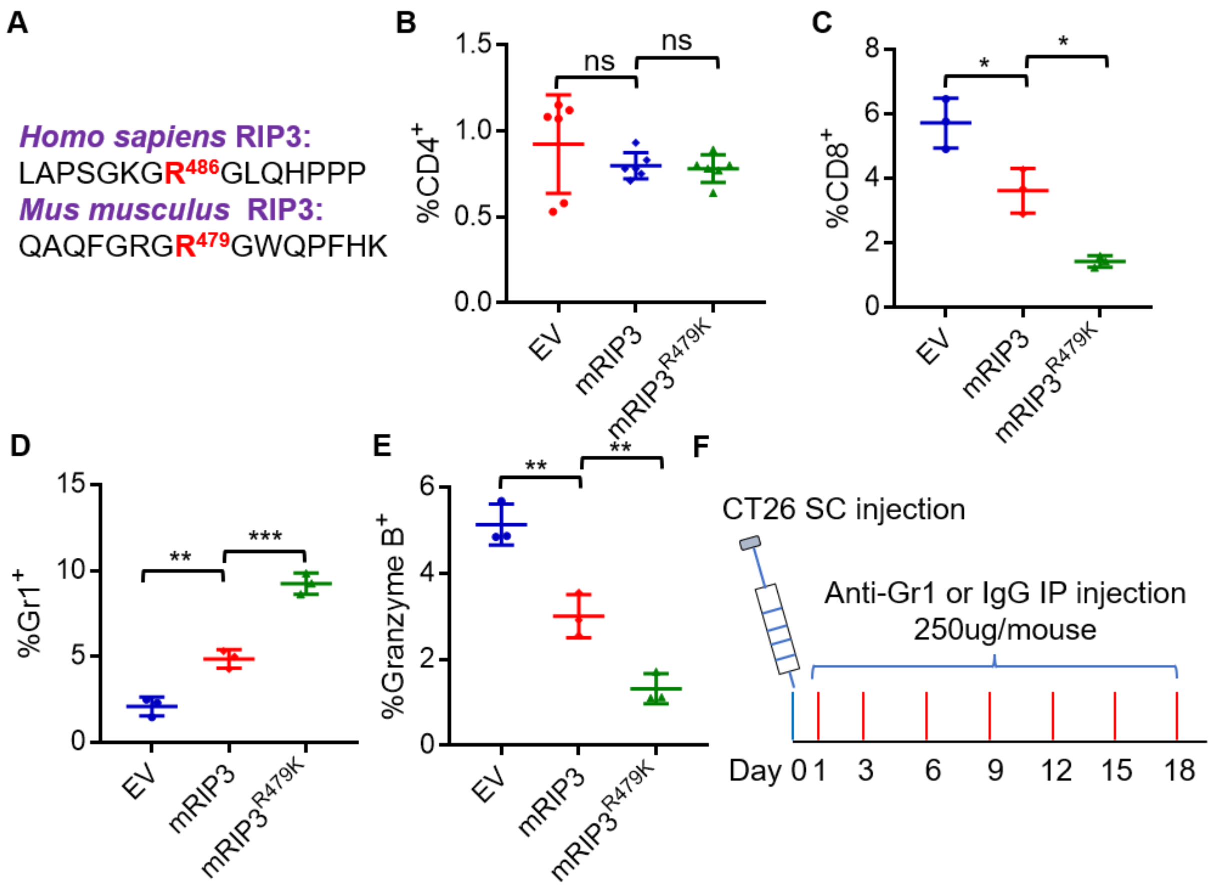

Figure S4 RIP3 methylation promotes anti-tumor immunity. (A) Sequence alignment identified murine RIP3 R479 as the homologous amino acid of human RIP3 R486. **(B)** Flowcytometry statistical analysis of $CD4^{+}$ T cell percentage in the control (EV), mRIP3, $mRIP3^{R479K}$ stable overexpression CT26 tumors. n=6. ns, not significant. **(C)** Immunohistochemistry statistical analysis of $CD8^{+}$ T cell percentage in the control (EV), mRIP3, $mRIP3^{R479K}$ stable overexpression CT26 tumors. n=3. *P<0.05, *P<0.05. **(D)** Immunohistochemistry statistical analysis of Gr^{+} MDSC percentage in the control (EV), mRIP3, $mRIP3^{R479K}$ stable overexpression CT26 tumors. n=3. **P<0.01, ***P<0.001. **(E)** Immunohistochemistry statistical analysis of Granzyme B^{+} cytotoxic cell percentage in the control (EV), mRIP3, $mRIP3^{R479K}$ stable overexpression CT26 tumors. n=3. **P<0.01, ** P <0.01. **(F)** *In vivo* treatment of anti-Gr1 antibody was performed by intraperitoneal (IP) injection with the dose of 250ug/mouse at day 1,3, 6, 9, 12, 15, 18 after subcutaneously (SC) tumor inoculation.

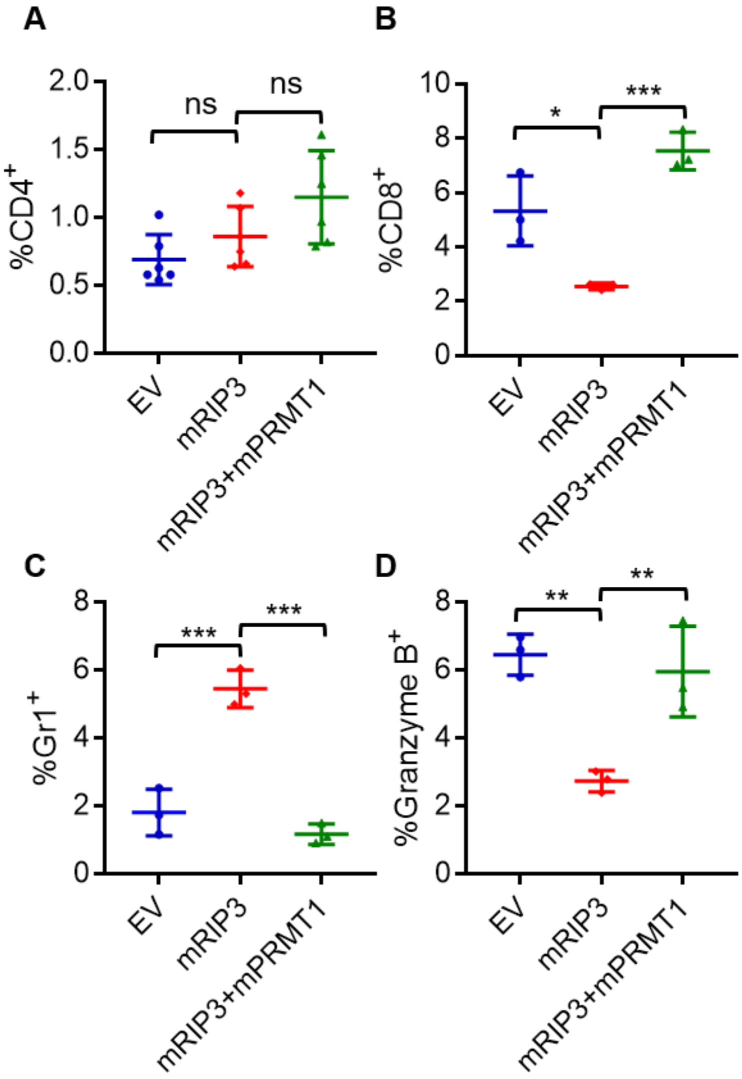

Figure S5 PRMT1 promotes anti-tumor immunity. (**A**) Flowcytometry statistical analysis of $CD4^+$ T cell percentage in the control (EV), mRIP3, mRIP3+mPRMT1 stable overexpression CT26 tumors. n=6. ns, not significant. (**B**) Immunohistochemistry statistical analysis of $CD8^+$ T cell percentage in the control (EV), mRIP3, mRIP3+mPRMT1 stable overexpression CT26 tumors. n=3. **P*<0.05, ****P*<0.001. (**C**) Immunohistochemistry statistical analysis of Gr^+ MDSC percentage in the control (EV), mRIP3, mRIP3+mPRMT1stable overexpression CT26 tumors. n=3. ****P*<0.001, ****P*<0.001. (**D**) Immunohistochemistry statistical analysis of Granzyme B^+ cytotoxic cell percentage in the control (EV), mRIP3, mRIP3/mPRMT1 stable overexpression CT26 tumors. n=3. ***P*<0.01, ***P*<0.01.

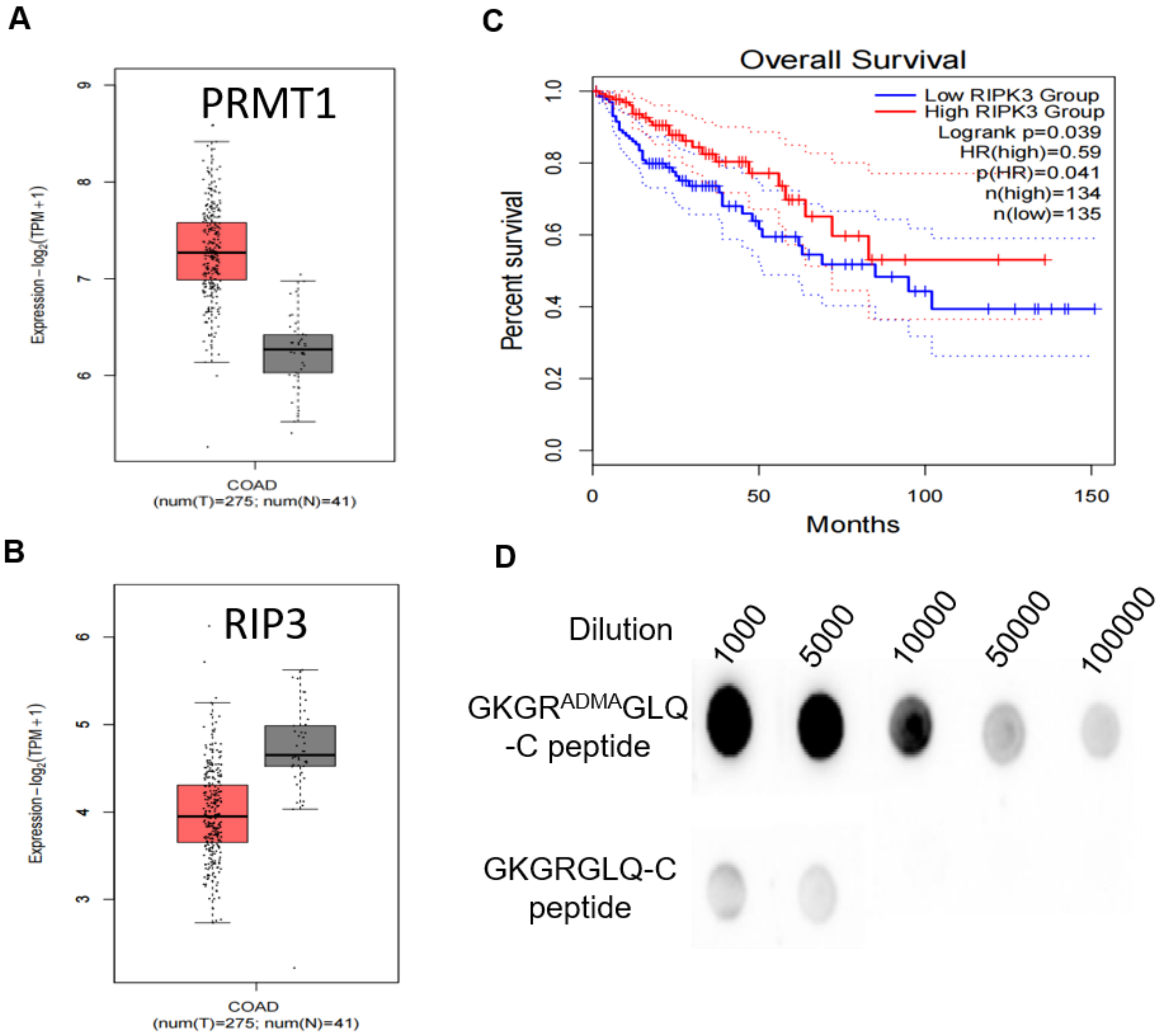

Figure S6 Clinical analysis of PRMT1 and RIP3 methylation in colon cancer patient samples. (**A**) GEPIA analysis of PRMT1 mRNA expression in colon carcinoma tissues and adjacent normal tissues in TCGA database. T, tumor tissues; N， adjacent normal tissues. (**B**) GEPIA analysis of RIP3 mRNA expression in colon carcinoma tissues and adjacent tissues in TCGA database. T, tumor tissues; N， adjacent normal tissues. (**C**) GEPIA overall survival analysis of colon carcinoma patients with low and high RIP3 mRNA expression in TCGA database. (**D**)Dot blot verification of RIP3 R486 methylation specific antibody $RIP3^{ADMA}$.

Table S1.

List of colon cancer patients.

Patient No.	Live-0, Dead-1	Live months	Gender	Age	T	N	M	Stage
1	1	13	Female	47	T4b	N0	M0	2
2	1	12	Male	65	T4b	N2	M0	3
3	0	68	Male	71	T3	N0	M0	2
4	0	68	Male	58	T3	N0	M0	2
5	1	17	Male	49	T3	N0	M1	4
6	0	68	Male	72	T2	N0	M0	1
7	0	68	Male	57	T4a	N1	M0	3
8	1	16	Male	83	T3		M0	
9	1	27	Female	66	T3		M0	
10	1	37	Female	60	T3	N1	M0	3
11	1	26	Male	47	T4b	N1	M1	4
12	1	25	Male	57	T3	N0	M1	4
13	0	68	Female	68	T3	N0	M0	2
14	0	68	Male	59	T3	N0	M0	2
15	1	14	Female	58	T3	N2	M0	3
16	1	8	Male	81	T3	N1	M0	3
17	1	8	Female	81	T3	N0	M0	2
18	0	67	Male	60	T3	N0	M0	2
19	1	24	Male	22	T3	N1	M0	3
20	1	31	Female	80	T3	N2	M0	3
21	1	29	Male	84	T3	N0	M0	2
22	1	65	Male	78	T3	N0	M0	2
23	1	56	Male	82	T3	N0	M0	2
24	0	67	Male	53	T3	N0	M0	2
25	1	42	Male	83	T3	N0	M0	2
26	1	5	Male	57	T3	N1	M0	3
27	1	23	Female	64	T4b	N0	M0	2
28	0	67	Female	63	T4a	N0	M0	2
29	0	67	Male	53	T3	N1	M0	3
30	1	47	Female	81	T3	N1	M0	3
31	0	67	Male	74	T4a	N0	M0	2
32	0	66	Male	60	T3	N0	M0	2
33	0	66	Female	78	T3	N0	M0	2
34	1	18	Female	55	T3	N1	M0	3
35	0	66	Female	51	T3	N0	M0	2
36	1	16	Female	53	T4a	N0	M0	2
37	1	20	Male	56	T3	N2	M0	3
38	1	45	Male	68	T3	N1	M0	3
39	1	25	Female	62	T3	N2	M0	3

40	1	23	Male	77	T3	N0	M0	2
41	0	65	Male	45	T2	N0	M0	1
42	1	34	Male	79	T4a	N0	M0	2
43	0	65	Female	41	T3	N1	M0	3
44	0	65	Male	82	T3	N0	M0	2
45	1	1	Male	70	T3	N1	M0	3
46	0	65	Male	46			M0	
47	1	46	Female	78	T3	N1	M1	4
48	1	26	Male	59	T3	N1	M0	3
49	0	65	Male	52	T3	N0	M0	2
50	0	65	Male	69	T3	N0	M0	2
51	1	27	Female	75	T4b	N2	M0	3
52	0	65	Male	70	T3	N1	M0	3
53	1	25	Male	72	T3	N0	M0	2
54	1	42	Female	61	T3	N1	M0	3
55	0	65	Male	78	T3	N1	M0	3
56	0	65	Male	73	T2	N0	M0	1
57	1	33	Male	85	T2	N0	M0	1
58	1	14	Female	56	T3	N0	M0	2
59	0	64	Male	45	T3	N1	M0	3
60	1	30	Female	86	T3	N1	M0	3
61	0	64	Male	76	T3	N0	M0	2
62	1	15	Female	71	T4b	N1	M0	3
63	1	32	Male	80	T3	N1	M0	3
64	0	64	Female	70		N0	M0	
65	1	16	Male	68	T3	N1	M0	3
66	0	64	Male	50	T3	N0	M0	2
67	0	64	Female	70	T3	N0	M0	2
68	0	63	Male	56		N0	M0	
69	0	63	Male	85	T3	N0	M0	2
70	0	63	Female	71		N0	M0	
71	0	63	Female	77		N1	M0	3
72	0	63	Female	61	T4a	N0	M0	2
73	0	63	Female	60	T2		M0	
74	1	17	Male	80	T3	N0	M0	2
75	0	63	Female	84	T3	N0	M0	2
76	0	63	Female	63	T3	N0	M0	2
77	1	1	Female	81		N1	M0	3
78	1	62	Female	87	T3	N0	M0	2
79	0	63	Male	58	T3	N0	M0	2
80	1	2	Female	58		N1	M0	3
81	0	62	Female	53	T4b		M0	
82	1	15	Female	93	T4b	N0	M0	2
83	0	62	Female	22	T3	N1	M0	3
84	1	27	Female	87	T4a	N1	M0	3

85	1	21	Male	82	T3	N2	M0	3
86	1	48	Male	80		N0	M0	
87	1	48	Female	85	T3	N0	M0	2
88	1	5	Male	55	T3	N2	M0	3
89	1	7	Female	76	T4b	N2	M0	3
90	1	5	Male	64	T3	N0	M0	2
91	1	67	Female	64	T3	N0	M0	2
92	1	18	Female	67	T3	N0	M0	2
93	1	12	Male	80	T3	N1	M0	3
