# Supplementary material for: PRMT1 reverts the immune escape of necroptotic colon cancer through RIP3 methylation
Source: Cell Death Dis. 2023 Apr 1;14(4):233. doi: 10.1038/s41419-023-05752-w (PMC10067857; doi:10.1038/s41419-023-05752-w)
Supplement: Supplementary file 2 — Supplemental material_uncropped figures [file 41419_2023_5752_MOESM2_ESM.pdf]

Fig. 1A

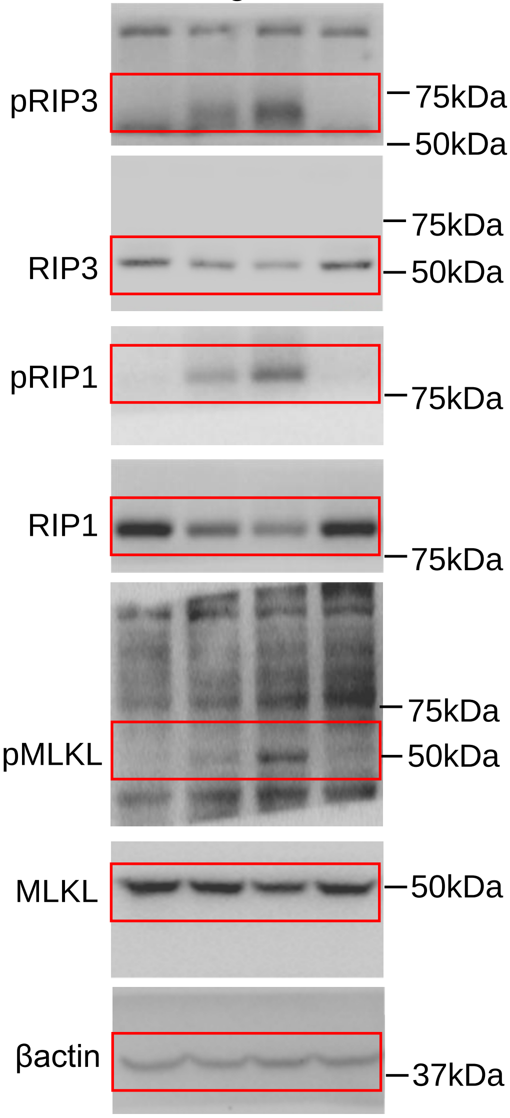

Fig. 1B

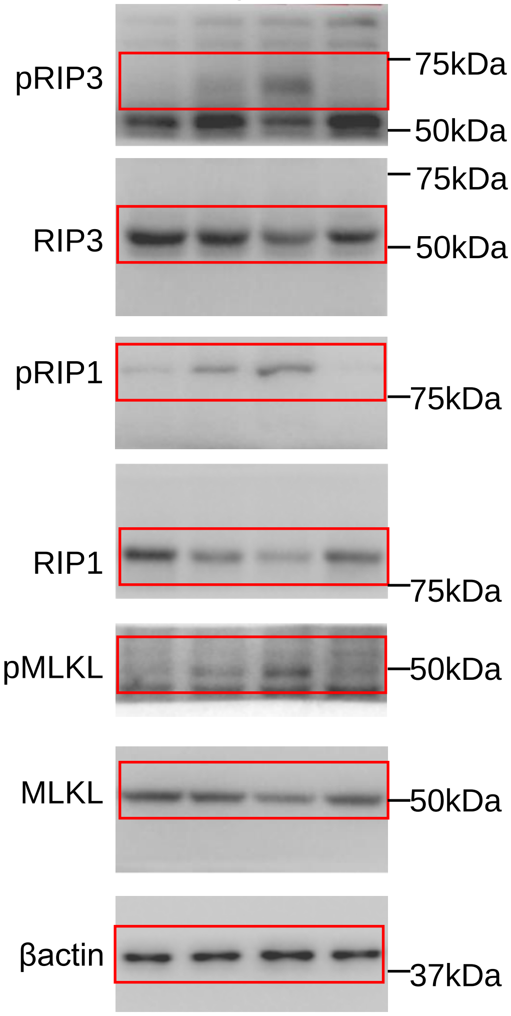

Fig. 1D

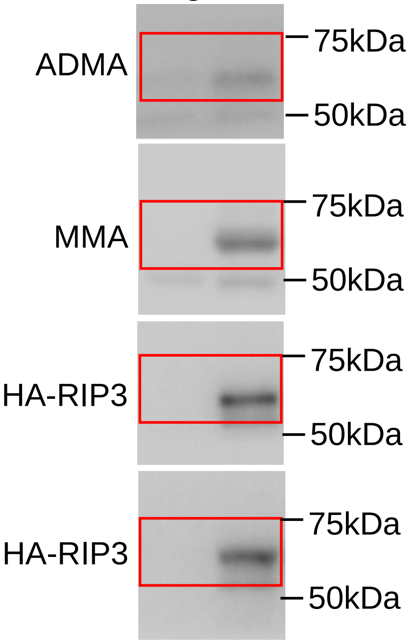

Fig. 1E

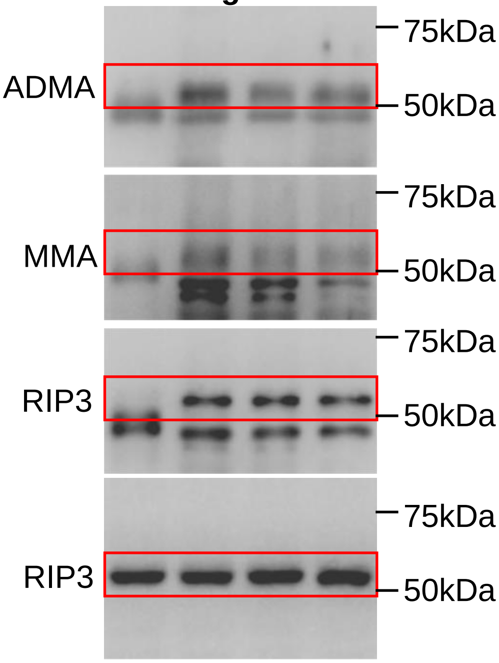

Fig. 1I

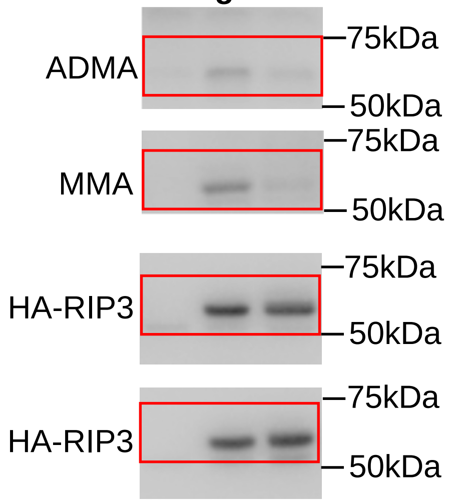

Fig. 1J

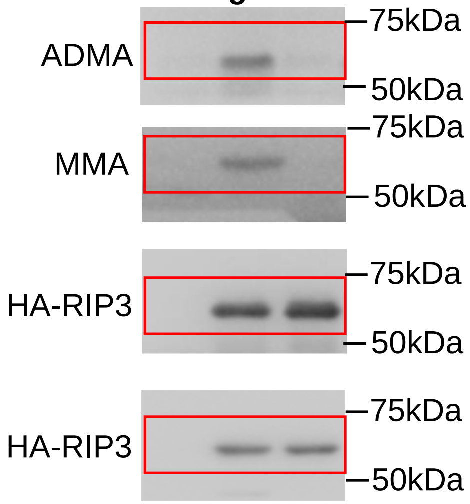

Fig. 1K

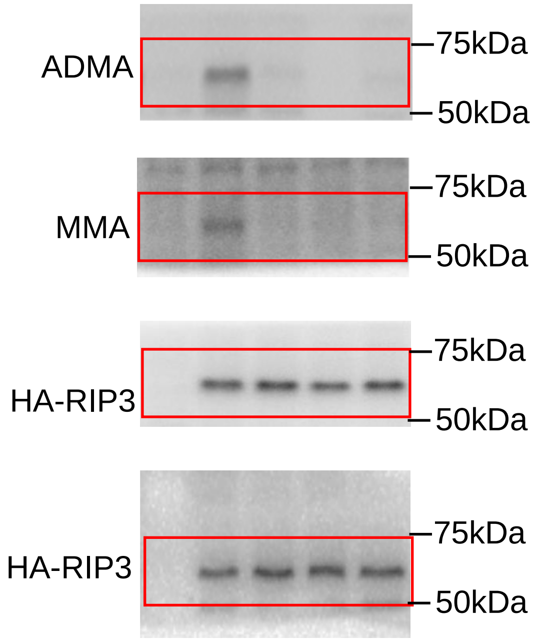

Fig. S1A

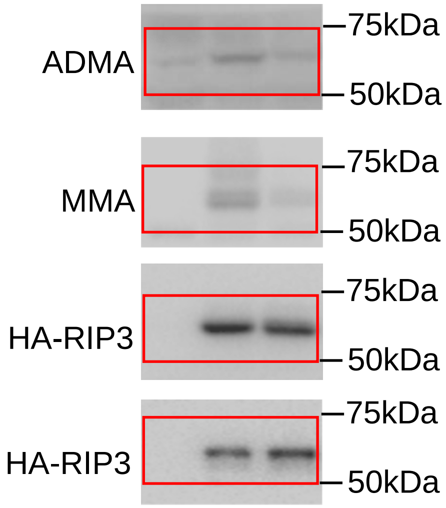

Fig. S1B

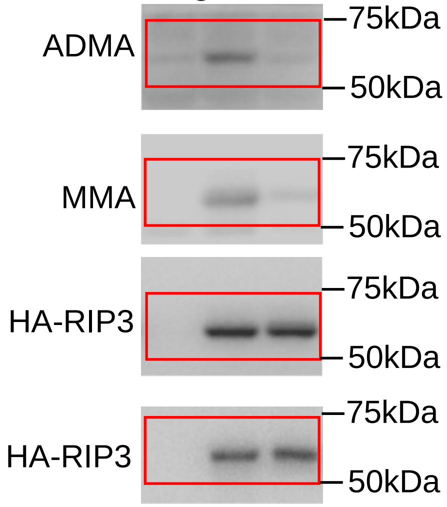

Fig. 2A

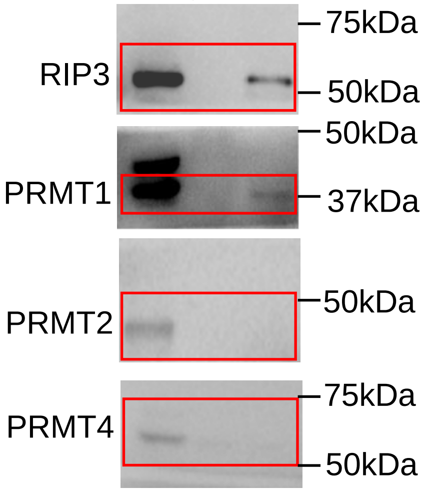

Fig. 2B

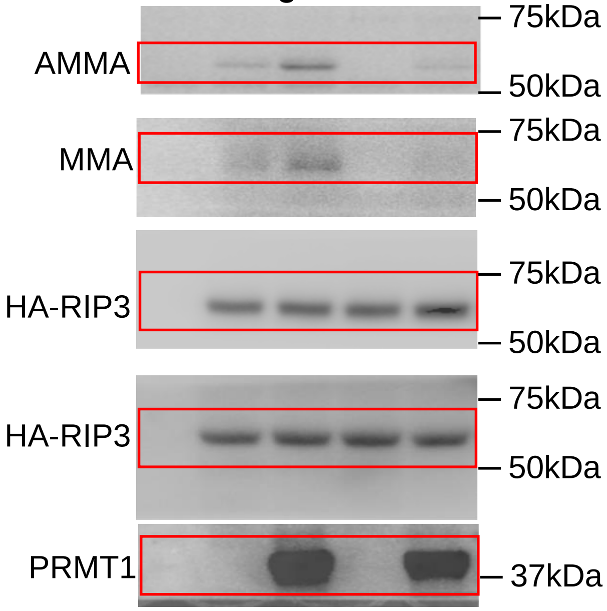

Fig. 2C

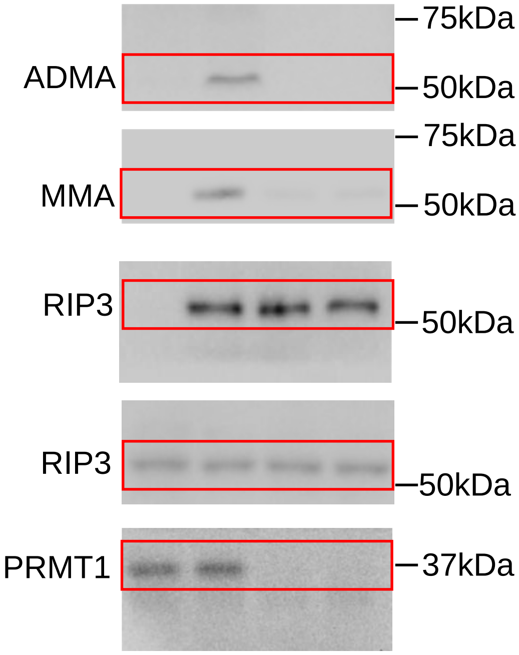

Fig. 2D

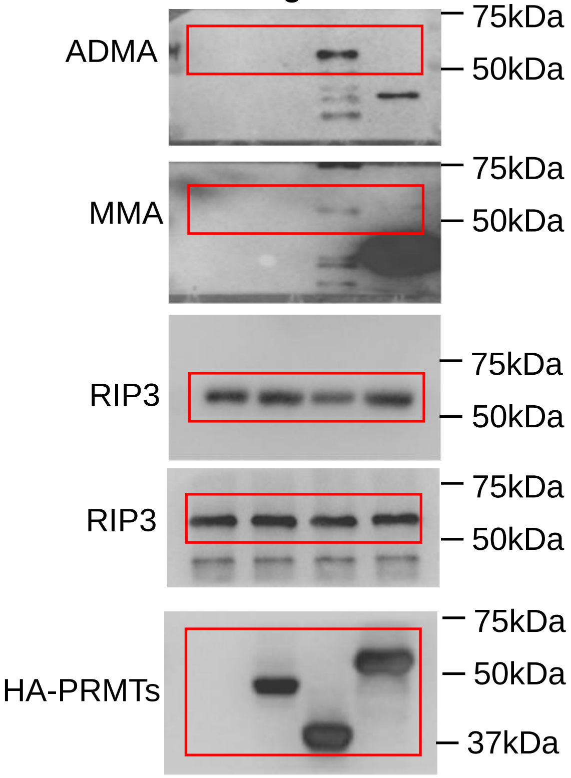

Fig. 2E

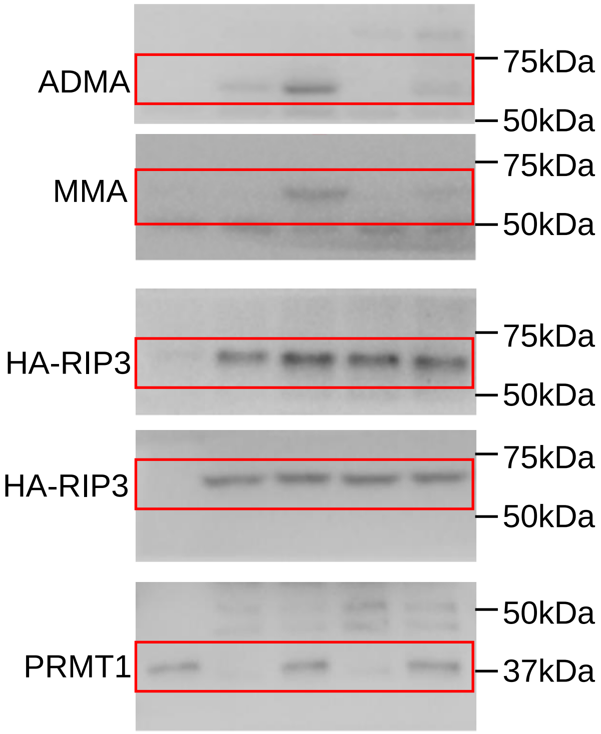

Fig. 2F

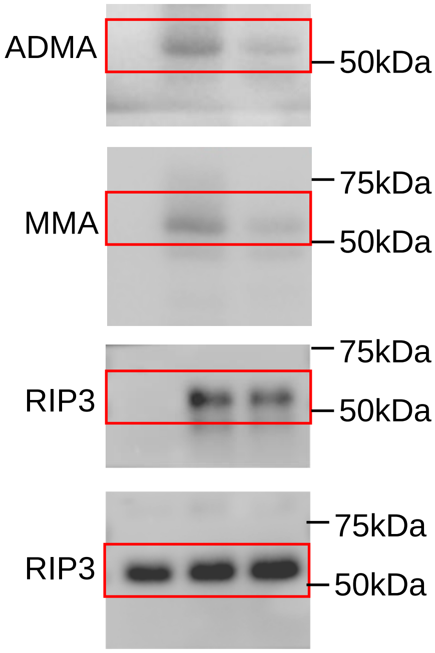

Fig. 2G

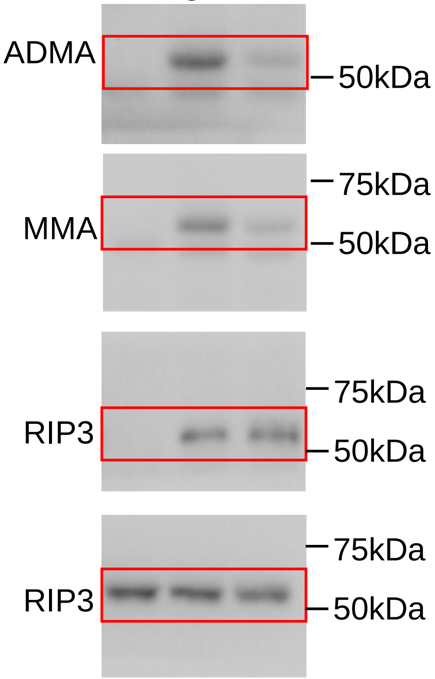

Fig. 3E

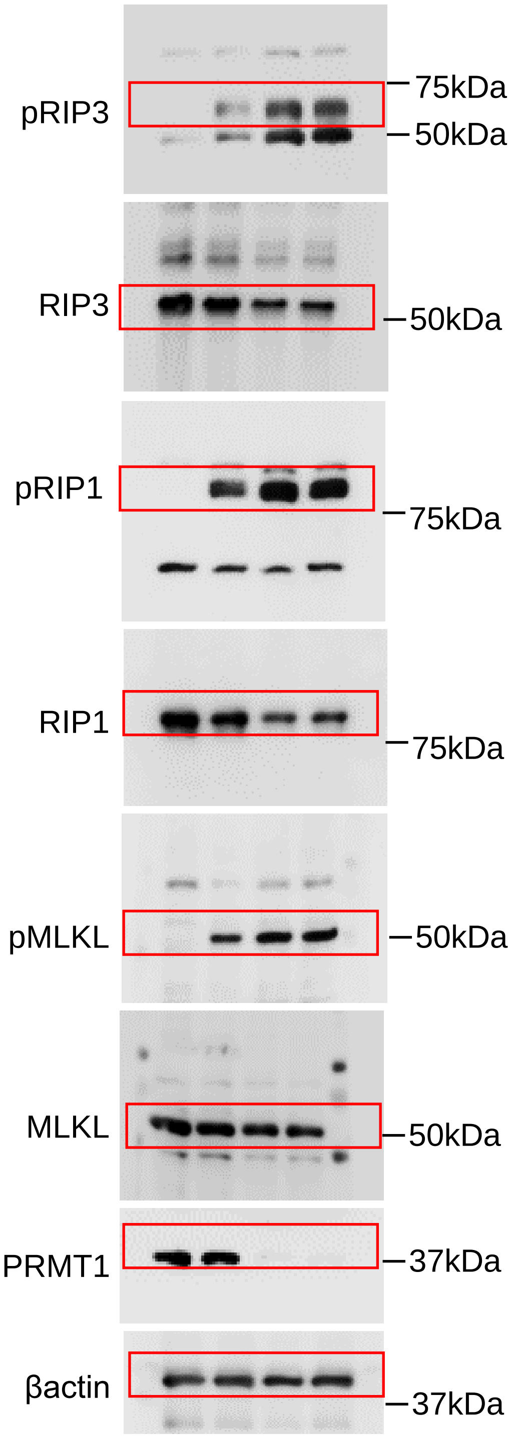
pRIP3
75kDa
50kDa
RIP3
50kDa
pRIP1
75kDa
RIP1
75kDa
pMLKL
50kDa
MLKL
50kDa
PRMT1
37kDa
βactin
37kDa

Fig. 3F

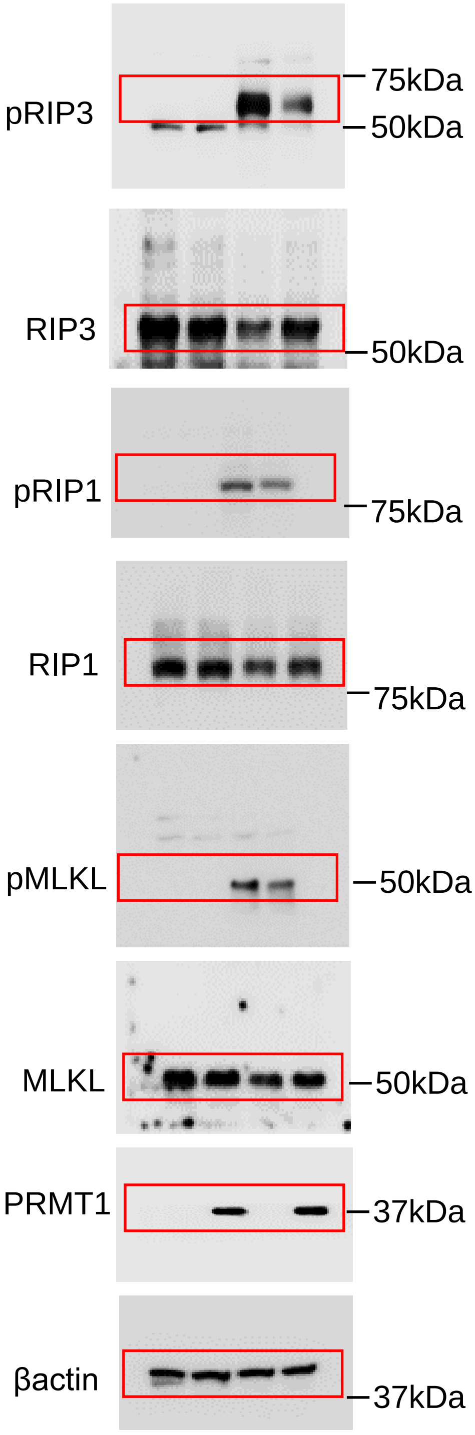
pRIP3
75kDa
50kDa
RIP3
50kDa
pRIP1
75kDa
RIP1
75kDa
pMLKL
50kDa
MLKL
50kDa
PRMT1
37kDa
βactin
37kDa

Fig. 3G

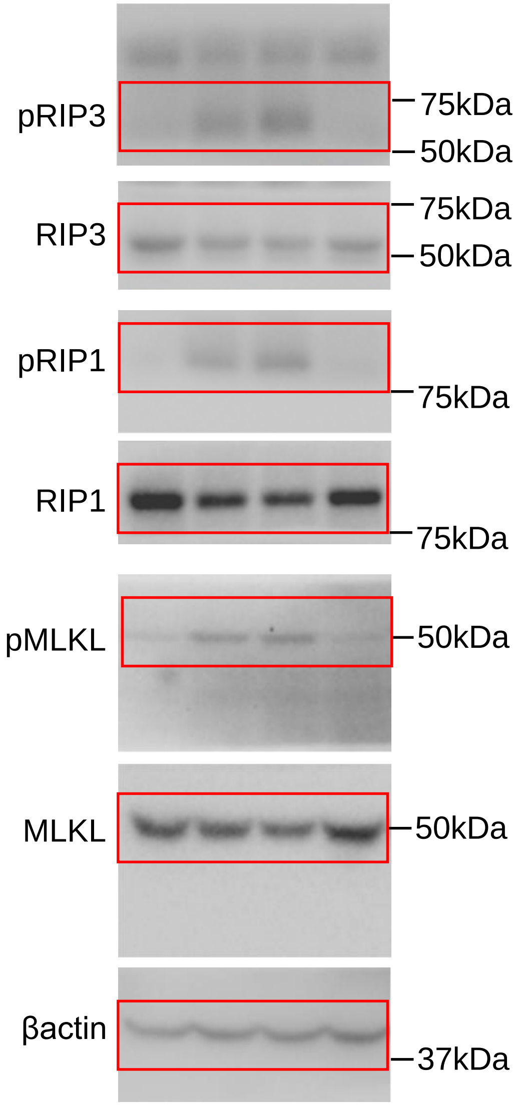

pRIP3
75kDa
50kDa
RIP3
75kDa
50kDa
pRIP1
75kDa
RIP1
75kDa
pMLKL
50kDa
MLKL
50kDa
βactin
37kDa

Fig. 3H

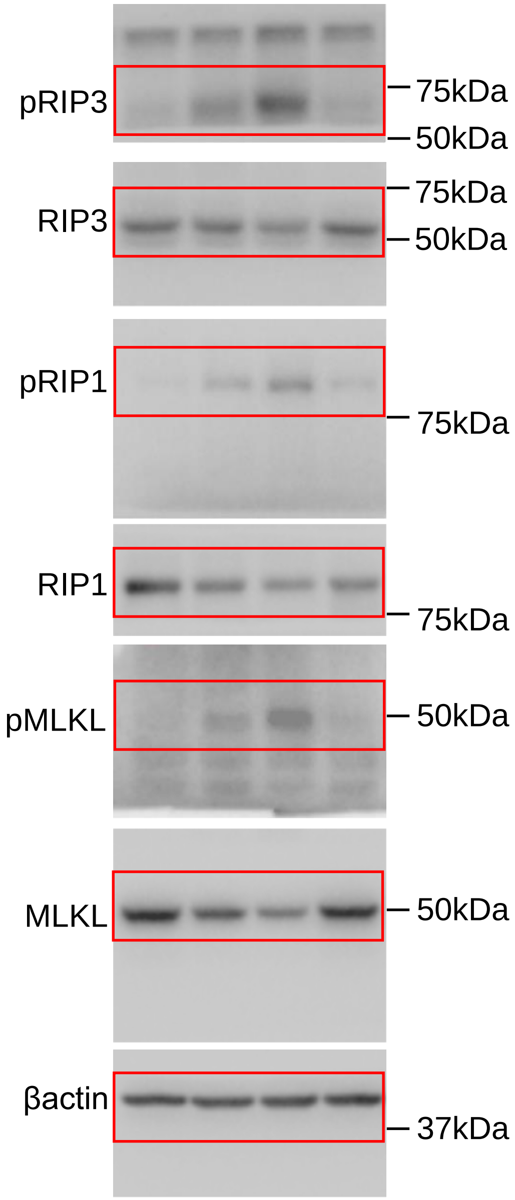

pRIP3
75kDa
50kDa
RIP3
75kDa
50kDa
pRIP1
75kDa
RIP1
75kDa
pMLKL
50kDa
MLKL
50kDa
βactin
37kDa

Fig. 3K

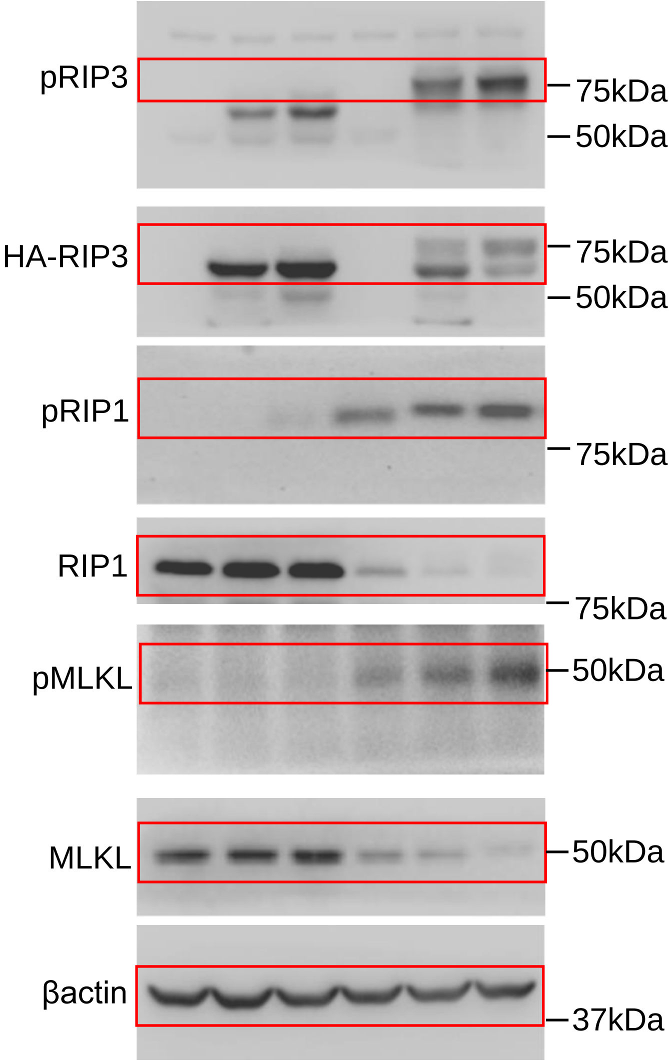
pRIP3
75kDa
50kDa
HA-RIP3
75kDa
50kDa
pRIP1
75kDa
RIP1
75kDa
pMLKL
50kDa
MLKL
50kDa
βactin
37kDa

Fig. 4A

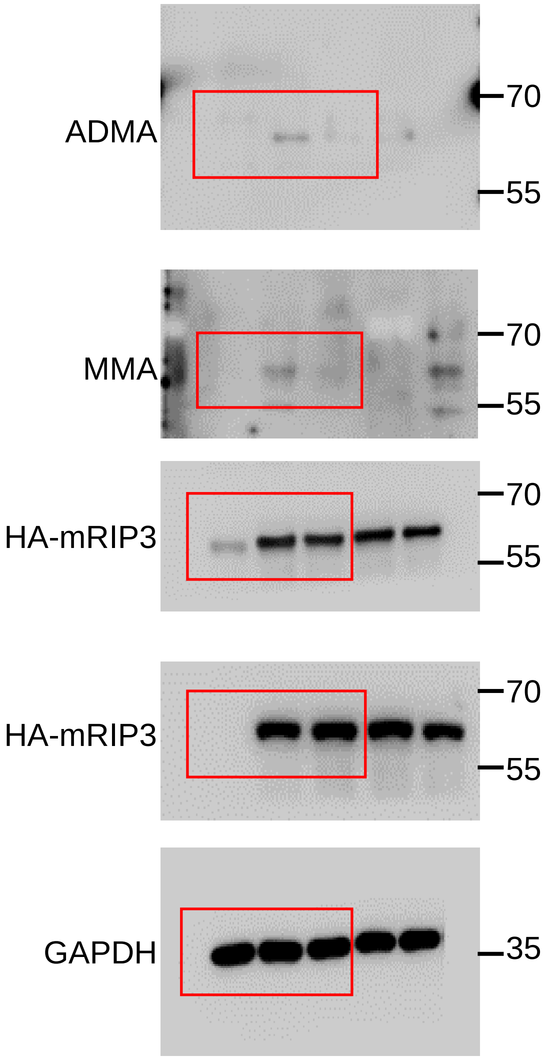
ADMA
70
55
MMA
70
55
HA-mRIP3
70
55
HA-mRIP3
70
55
GAPDH
35

Fig. 4B

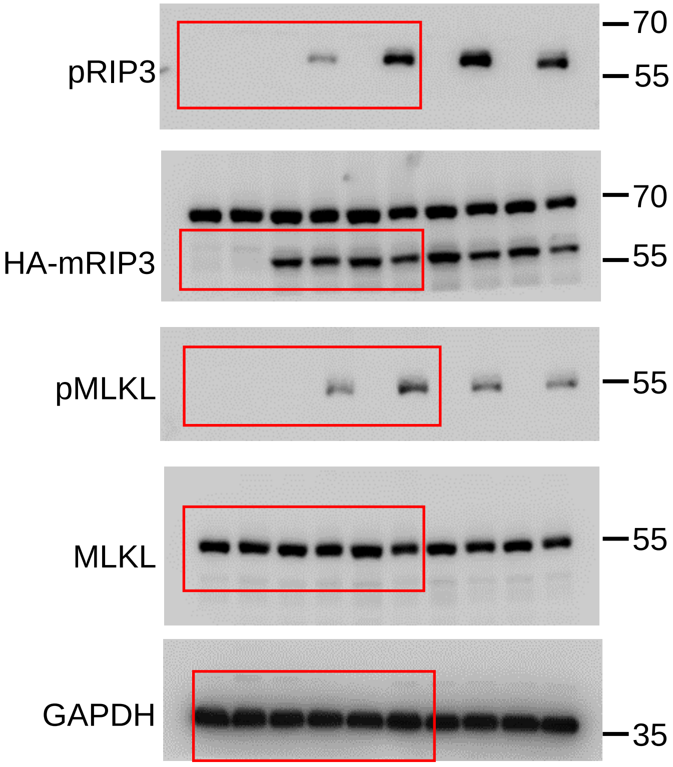
pRIP3
70
55
HA-mRIP3
70
55
pMLKL
55
MLKL
55
GAPDH
35

Fig. 5A

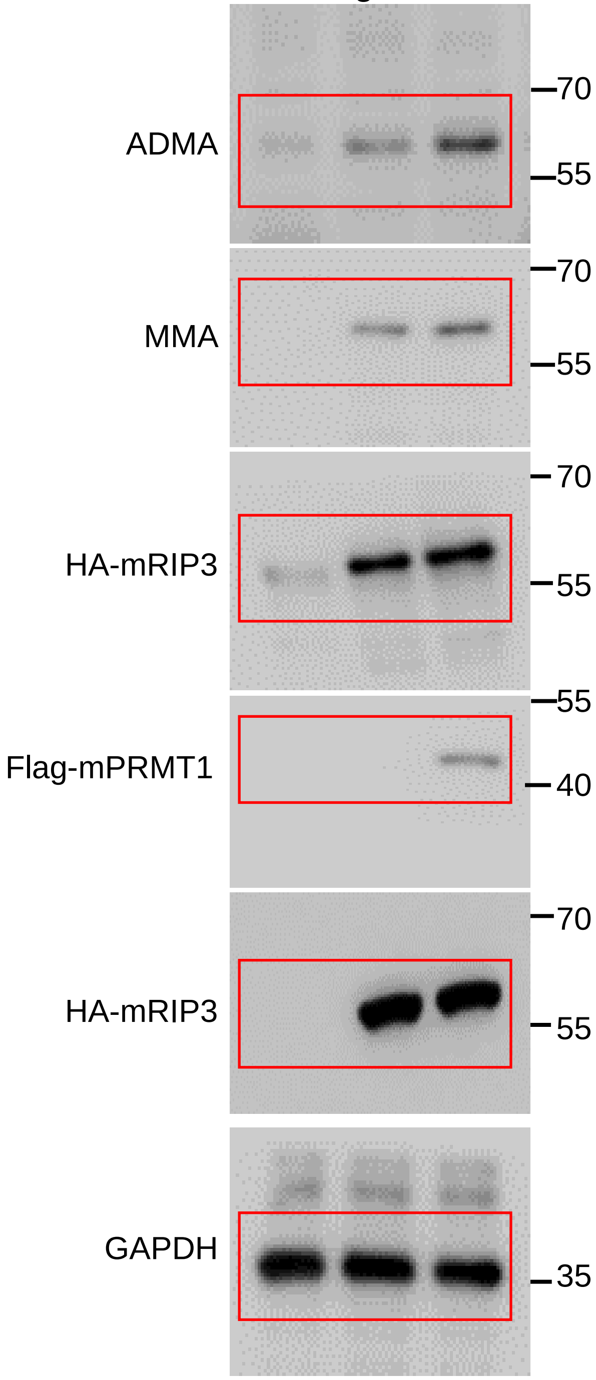
ADMA
70
55
MMA
70
55
HA-mRIP3
70
55
Flag-mPRMT1
55
40
HA-mRIP3
70
55
GAPDH
35

Fig. 5B

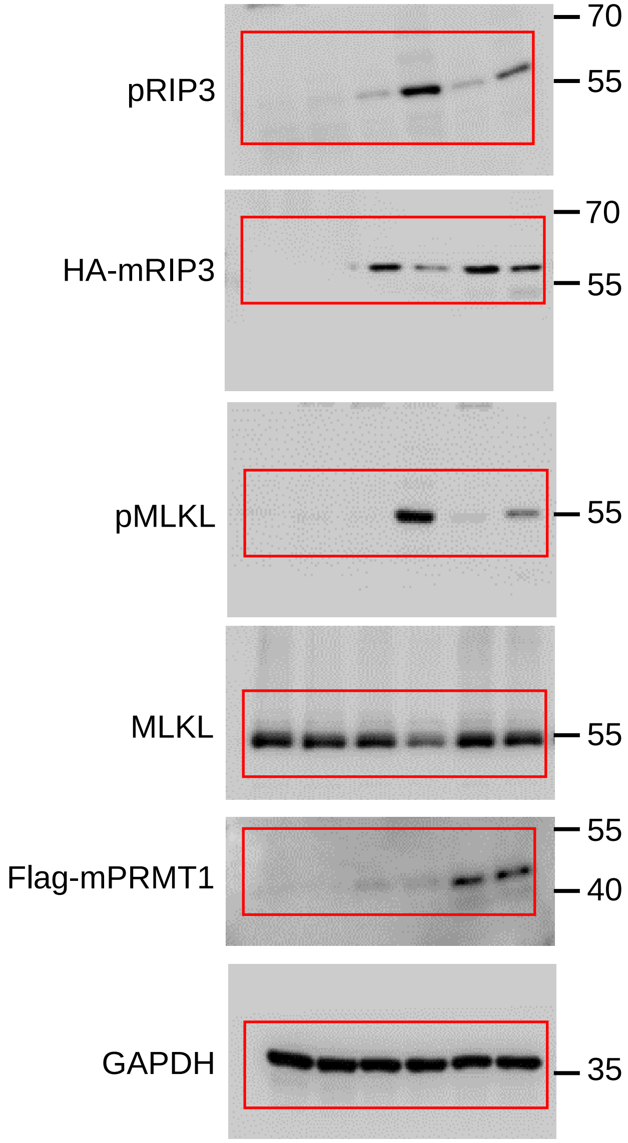
pRIP3
70
55
HA-mRIP3
70
55
pMLKL
55
MLKL
55
Flag-mPRMT1
55
40
GAPDH
35

Fig. 6B

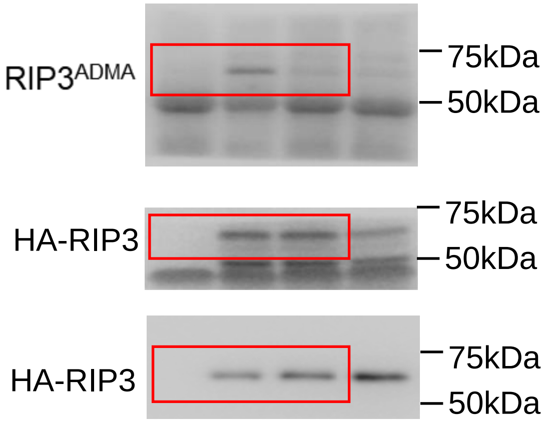

RIP3ADMA
75kDa
50kDa
HA-RIP3
75kDa
50kDa
HA-RIP3
75kDa
50kDa

Fig. 6C

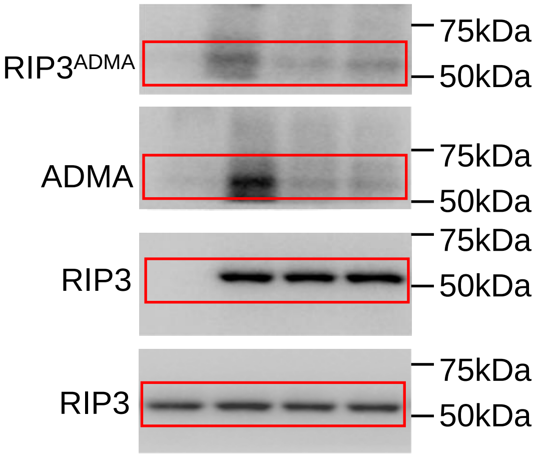

RIP3ADMA
75kDa
50kDa
ADMA
75kDa
50kDa
RIP3
75kDa
50kDa
RIP3
75kDa
50kDa
